# A Rapid and Efficient Screening System for Neutralizing Antibodies and Its Application for SARS-CoV-2

**DOI:** 10.3389/fimmu.2021.653189

**Published:** 2021-03-22

**Authors:** Xiaojian Han, Yingming Wang, Shenglong Li, Chao Hu, Tingting Li, Chenjian Gu, Kai Wang, Meiying Shen, Jianwei Wang, Jie Hu, Ruixin Wu, Song Mu, Fang Gong, Qian Chen, Fengxia Gao, Jingjing Huang, Yingyi Long, Feiyang Luo, Shuyi Song, Shunhua Long, Yanan Hao, Luo Li, Yang Wu, Wei Xu, Xia Cai, Qingzhu Gao, Guiji Zhang, Changlong He, Kun Deng, Li Du, Yaru Nai, Wang Wang, Youhua Xie, Di Qu, Ailong Huang, Ni Tang, Aishun Jin

**Affiliations:** ^1^ Department of Immunology, College of Basic Medicine, Chongqing Medical University, Chongqing, China; ^2^ Chongqing Key Laboratory of Basic and Translational Research of Tumor Immunology, Chongqing Medical University, Chongqing, China; ^3^ Key Laboratory of Medical Molecular Virology, Department of Medical Microbiology and Parasitology, School of Basic Medical Sciences, Shanghai Medical College, Fudan University, Shanghai, China; ^4^ Key Laboratory of Molecular Biology on Infectious Diseases, Ministry of Education, Chongqing Medical University, Chongqing, China; ^5^ Department of Breast Surgery, Harbin Medical University Cancer Hospital, Harbin, China; ^6^ Department of Pediatrics, Yongchuan Hospital Affiliated to Chongqing Medical University, Chongqing, China; ^7^ Department of Laboratory, The Third Affiliated Hospital of Chongqing Medical University, Chongqing, China

**Keywords:** SARS-CoV-2, neutralizing antibodies, methodology, spike protein, receptor-binding domain (RBD)

## Abstract

After the pandemic of COVID-19, neutralizing antibodies (NAbs) against SARS-CoV-2 have been developed for the prophylactic and therapeutic purposes. However, few methodologies are described in detail on how to rapidly and efficiently generate effective NAbs to SARS-CoV-2. Here, we integrated and optimized a strategically screening method for NAbs, which has enabled us to obtain SARS-CoV-2 receptor-binding domain (RBD) specific NAbs within 6 days, followed by additional 9 days for antibody production and function analysis. Using this method, we obtained 198 specific Abs against SARS-CoV-2 RBD from the blood samples of COVID-19 convalescent patients, and 96 of them showed neutralizing activity. At least 20% of these NAbs exhibited advanced neutralizing potency and high affinity, with the top two NAbs showing half-maximal inhibitory concentration (IC_50_) to block authentic SARS-CoV-2 at 9.88 and 11.13 ng/ml, respectively. Altogether, our study provides an effective methodology with high applicable value for discovering potential preventative and therapeutic NAbs for the emerging infectious diseases.

## Introduction

In the past two decades, pandemic outbreaks of three novel pathogenic human coronaviruses, severe acute respiratory syndrome coronavirus (SARS-CoV), Middle East respiratory syndrome coronavirus (MERS-CoV) and SARS-CoV-2, have caused high mortality and unprecedented social and economic consequences (1–4). While vaccines are effective in preventing infectious diseases, NAbs are an alternative treatment strategy for blocking virus infection. During the outbreaks of SARS-CoV and MERS-CoV, convalescent patients’ plasma containing NAbs was a safe and effective treatment option to reduce mortality in severe cases (5, 6). However, convalescent plasma is limited and the polyclonal non-neutralizing Abs in the plasma may cause undesired side effects (7). The NAb therapeutics is an effective replacement of convalescent plasma therapy. Therefore, a rapid and efficient screening method of desired NAbs is greatly needed.

The viral spike (S) of SARS-CoV-2 containing the receptor-binding domain (RBD) is responsible for the viral entry into host cells (8, 9). To date, some potent NAbs to SARS-CoV-2 have been promptly developed by different teams, with various methodologies employed for the screening of NAbs (10–23). Some studies utilized SARS-CoV-2 S- or RBD-labeled memory B cells from COVID-19 convalescent patients, and directly amplified Ab genes by RT-PCR and nested PCR at a single-cell level (17, 21). Others activated and expanded plasma cells with stimulators and cytokines *in vitro* to select NAbs (11, 12). Also, humanized mice were used to generate full human mAbs against S protein (10, 23). Furthermore, the single-cell sequencing technology was applied in combination with the enrichment of antigen-specific B cells, which led to the quick detection of thousands of antigen specific mAbs sequences (14, 15). Although these studies showed that NAbs against SASR-CoV-2 could be obtained from convalescence patients, the details of methodologies and the feasibilities of advancing NAb screening efficiency remain unclear.

Here, we presented strategically optimized system for the fast screening of fully human monoclonal Abs with neutralizing capability in, as short as, 6 days. A total of 198 Abs against RBD of SARS-CoV-2 were obtained with this method, and 50% of them were potent neutralizing Ab candidates in a preliminary screening with SARS-CoV-2 pseudovirus. Furthermore, we obtained 20 potent NAbs against authentic SARS-CoV-2 with high affinity to the RBD. Among them, the top 2 NAbs exhibit IC_50_ values around 10 ng/ml to block authentic SARS-CoV-2. Therefore, this screening system can efficiently generate a large number of potent NAbs, which facilitate the development of NAbs candidate biologics for treatment and prevention of infectious diseases, and can also applied for the development of monoclonal Ab drugs for tumor immunotherapy.

## Materials And Methods

### Isolation of Single RBD-Specific Memory B Cells by FACS

PBMCs from the convalescent patients were thawed and cultured with RPMI-1640 medium, supplemented with 10% fetal bovine serum (FBS), at 37°C in a 5% CO_2_ humidified incubator for 24 hours. The staining method for the mixed samples (approximately 6 x10^7^ cells) was as follows: 2 μg/ml RBD-his in 200 μl PBS (added with 2% FBS) was mixed with the specific antibody cocktail required for staining B cell. Then these PBMCs was incubated with a mixed antibodies cocktail at 4°C for 30 min (the antibodies cocktail including FITC-anti-human CD19 antibody (Biolegend, clone: SJ25C1), BV421-anti-human IgD antibody (Biolegend, clone: IA6-2), PerCP-Cy5.5-anti-human IgG antibody (Biolegend, clone: M1310G05), APC-anti-his tag antibody (Biolegend, clone: J095G46)). Dead dye (LIVE/DEAD™ Fixable Near-IR Dead Cell Stain Kit, Thermo Fisher) was added at 4°C for 20 min. After washing the cells, the FACS analysis was performed by BD FACSAria III Cell Sorter (BD Biosciences) with CD19 versus SSC-A gating CD19^+^ population, FSC-A versus FSC-H excluding doublets, FSC-A versus SSC-A identifying cell population. Then CD19 versus Dead Dye was gated to remove dead cells. RBD-specific single memory B cells were gated by CD19^+^IgD^-^IgG^+^His^+^, and single-cell sorted into 96-well PCR plates (free of DNase and RNase, Bio-Rad). The Plates were stored at -80°C until BCR Cloning. Data analysis was performed utilizing the FlowJo software (FlowJo, LLC).

### Amplification of Single-Cell BCR Variable Region

Our primers for PCR were designed from leader sequences and J region sequence of immunoglobulin (Ig) annotated by the IMGT reference directory (http://www.imgt.org/vquest/refseqh.html). An adaptor sequence was added to the 5′ end of the leader primers for the 2^nd^ PCR. 31 leader primers (AP_G_leader Mix) was designed for the heavy chain of Ig, and 19 leader primers (AP_K_leader Mix) was used in the amplification of the kappa chain of Ig, and 21 leader primers (AP_L_leader Mix) for the lambda chain of Ig were designed. For the initial step of RT-PCR, 5 μl of the RT_Mix_A was added into each well of 96 well plates containing a single B cell. Then the mixture was incubated at 65°C for 5 min and put on ice immediately for 3 min. 5 μl RT_Mix_B was added into each well of the plate with reaction program: 45 °C for 45 min, 70 °C for 15 min. 1 μl of RT product was moved to the well of a new 96 well plate containing 9 μl 1st PCR Mix Gamma/Kappa/Lamda, respectively. The PCR program for 1st PCR: 95°C for 3 min, 30 cycles of 95°C for 10 sec, 55°C for 5 sec, and 72°C for 1 min. 1 μl of the tenfold-diluted 1st PCR product was then added into each well of a new 96 well plate holding 9 μl 2^nd^ PCR Mix Gamma/Kappa/Lamda, respectively. The PCR program for 2^nd^ PCR: 95°C for 3 min, 35 cycles of 95°C for 10 sec, 55°C for 5 sec, and 72°C for 45 sec. The second PCR products were further cloned into the antibody linear expression cassettes or expression vectors to express full IgG1 antibodies. PCR reaction Mixtures are prepared as described in **Supplementary Table 6**. All of the PCR primers are listed in **Supplementary Table 1** and prepared in **Supplementary Table 7**.

### Generation of Linear Antibody Expression Cassettes and Expression of Abs

2^nd^ PCR products were used to ligate with the expression cassettes directly by overlapping PCR. The products were purified with the ethanol precipitation method. Briefly, 120 μl of absolute ethanol and 6 μl of 3 M sodium acetate were mixed with 60 μl of the Overlap PCR product. Then the reagents were incubated at -80°C for 30 minutes. After centrifuging at 10,000 rpm for 20 minutes, the supernatant was discarded the and the pellet adhered on the tube were rinsed with 200 μl 70% ethanol and absolute ethanol and evaporated the ethanol at 56°C for 10 min. 40 μl sterile water was added to dissolve the DNA pellet. After measuring the nucleic acid concentration, purified overlapping PCR products of paired heavy and light chain expression cassettes were co-transfected in HEK293T cells, and then detected the expressions of IgG1 proteins in supernatants. The binding ability of transfected culture supernatants to SARS-CoV-2 RBD was tested by ELISA after 48 hours.

### Recombinant Antibody Production and Purification

For the construction of antibody expression vectors, antibody cDNA encoding the entire constant region of immunoglobulin gamma-1 heavy chain (Cγ) (GenBank accession number: BC073782.1) and light chain, kappa chain(Cκ)(GenBank accession number: JQ837832.1) or lambda chain (Cλ) (GenBank accession number: AY172962.1) were cloned into a pcDNA3.4 expression vector (Catalog No. A14697, ThermoFisher), respectively. We amplified antibody cDNAs for VH and VL fragments using a single-cell 5′-RACE method with primers for γ chain, κ chain and λ chains and inserted separately them into the linearized plasmids (pcDNA3.4) that encoded the constant regions cDNA for the heavy chains and light chains *via* a homologous recombination kit (Catalog No. C112, Vazyme). A pair of plasmids separately expressing heavy and light chain of antibodies were transiently co-transfected into Expi293™ cells (Catalog No. A14528, ThermoFisher) with ExpiFectamine™ 293 Reagent. Then the cells were cultured in a shaker incubator at 120 rpm and 8% CO_2_ at 37 °C. After 7 days, the supernatants with the secretion of antibodies were collected and captured by protein G Sepharose (GE Healthcare). The bound antibodies on the Sepharose were eluted and dialyzed into phosphate-buffered saline (PBS). The purified antibodies were used in following binding and neutralization analyses.

### ELISA Binding Assay and Competitive ELISA

2 μg/ml the recombinant S or RBD proteins derived from SARS-CoV-2, SARS-CoV, or MERS-CoV (Sino Biological, Beijing) were coated on 384-well plates (Corning) at 4°C overnight. Plates were blocked with blocking buffer (PBS containing 5% FBS and 2% BSA) at 37°C for 1 hour. Serially diluted convalescents’ plasma or mAbs were added into the plates and incubated at 37°C for 30 min. Plates were washed with phosphate-buffered saline, 0.05% Tween-20 (PBST) and ALP-conjugated goat anti-human IgG (H+L) antibody (Thermo Fisher) was added into each well and incubated at 37°C for 1 hour. Lastly, the PNPP substrate was added, and absorbance was measured at 405 nm by a microplate reader (Thermo Fisher). For a competitive ELISA to test the effect of mAbs on blocking ACE2 binding RBD, 2 μg/ml the recombinant ACE2 (Sino Biological, Beijing) was added in 384-well plates and overnight at 4°C, followed by blocking with the blocking buffer and washing. 0.5 μg/ml RBD-mouse-Ig-Fc was pre-incubated with purified mAbs at 37°C for 1 hour, followed by adding into the wells coated with ACE2 and incubated at 37°C for 1 hour. Unbound antigen was removed with washes. Then ALP-conjugated anti-mouse-Ig-Fc antibody was added into the wells and incubated at 37°C for 30 min. PNPP was added and measured as above.

### Pseudovirus Neutralization Assay

Pseudovirus was generated as previously described (24). HEK293T cells were transfected with psPAX2, pWPXL Luciferase, and pMD2.G plasmid encoding either SARS-CoV-2 S. The supernatants were harvested 48 hours later, filtered by 0.45 μm filter and centrifugated at 300 g for 10 min to collect the supernatant and then aliquoted and storied at -80°C. The HEK293T supernatants or the serial diluted antibodies were incubated with pseudovirus at 37°C for 1 hour. The mixture of viruses and specimens was then added in a hACE2 expressing cell line (hACE2-293T cell). After 48 hours culture, the luciferase activity of infected hACE2/293T cells was measured by the Bright-Luciferase Reporter Assay System (Promega). Relative luminescence unit (RLU) of Luc activity was detected using the ThermoFisher LUX reader. All experiments were performed at least three times and expressed as means ± standard deviations (SDs). Half-maximal inhibitory concentrations (IC_50_) were calculated using the four-parameter logistic regression in GraphPad Prism 8.0.

### Authentic SARS-CoV-2 Virus Neutralization Assays

An authentic SARS-CoV-2 neutralization assay was performed in a biosafety level 3 laboratory of Fudan University. Serially diluted mAbs were incubated with authentic SARS-CoV-2 (nCoV-SH01, GenBank: MT121215.1, 100 TCID50) at 37°C for 1 hour. After incubation, the mixtures were then transferred into 96-well plates, which were seeded with Vero E6 cells. After incubation at 37°C for 48 hours, each well was examined for CPE and supernatant viral RNA by RT-qPCR. For RT-qPCR, the viral RNA was extracted from the collected supernatant using Trizol LS (Invitrogen) and used as templates for RT-qPCR analysis by Verso 1-step RT-qPCR Kit (Thermo Scientific) following the manufacturer’s instructions. PCR primers targeting SARS-CoV-2 N gene (nt608-706) were as followed (forward/reverse): 5′-GGGGAACTTCTC CTGCTAGAAT-3′/5′-CAGACATTTTGCTCTCAAGCTG-3′. qRT-PCR was performed using the LightCycler 480 II PCR System (Roche) with the program as followed: 50°C 15 min; 95°C 15 min; 40 cycles of 95°C 15 sec, 50°C 30 sec, 72°C 30 sec.

### Antibody Binding Affinity Measurement by SPR

The affinity of antibody binding SARS-Cov-2-S-RBD was measured *via* the Biacore X100 platform. The CM5 chip (GE Healthcare) was coupled with an anti-human IgG-Fc antibody to capture 9000 RU antibodies. Gradient concentrations of SARS-Cov-2 RBD (Sino Biological Inc.) were diluted (2-fold dilution, from 50 nM to 0.78 nM) with HBS-EP^+^ Buffer (0.01 M HEPES, 0.15 M NaCl, 0.003 M EDTA and 0.05% (v/v) Surfactant P20, pH 7.4), then injected into the human IgG capturing chip. The sensor surface was regenerated with 3 M magnesium chloride at the end of each cycle. The affinity was calculated using a 1:1 binding fit model in Biacore X100 Evaluation software (Version:2.0.2).

### Sequence Analysis of Antigen-Specific mAb Sequences

IMGT/V-QUEST (http://www.imgt.org/IMGT_vquest/v quest) and Ig BLAST (https://www.ncbi.nlm.nih.gov/igblast/), MIXCR (https://mixcr.readthedocs.io/en/master/) and VDJ tools (https://vdjtools-doc.readthedocs.io/en/master/overlap.html) tools were used to do the VDJ analysis and annotation, germline divergence for each antibody clone. The Phylogeny tree analysis of IgG heavy and light chain variable genes was performed with Mega X (Molecular Evolutionary Genetics Analysis across computing platforms) by the Maximum Likelihood method. Abs DNA sequences were compared with each other by Clustal W (pairwise alignments) to analyze sequence similarity, and EvolView (https://www.evolgenius.info/evolview/) was used for the decoration of Phylogeny tree. R packages (ggplot2, p heatmap) were used for the bar chart, heatmap and Cicos plot.

## Results

### A Rapid and Efficient NAbs Screening System

**Figure 1:**
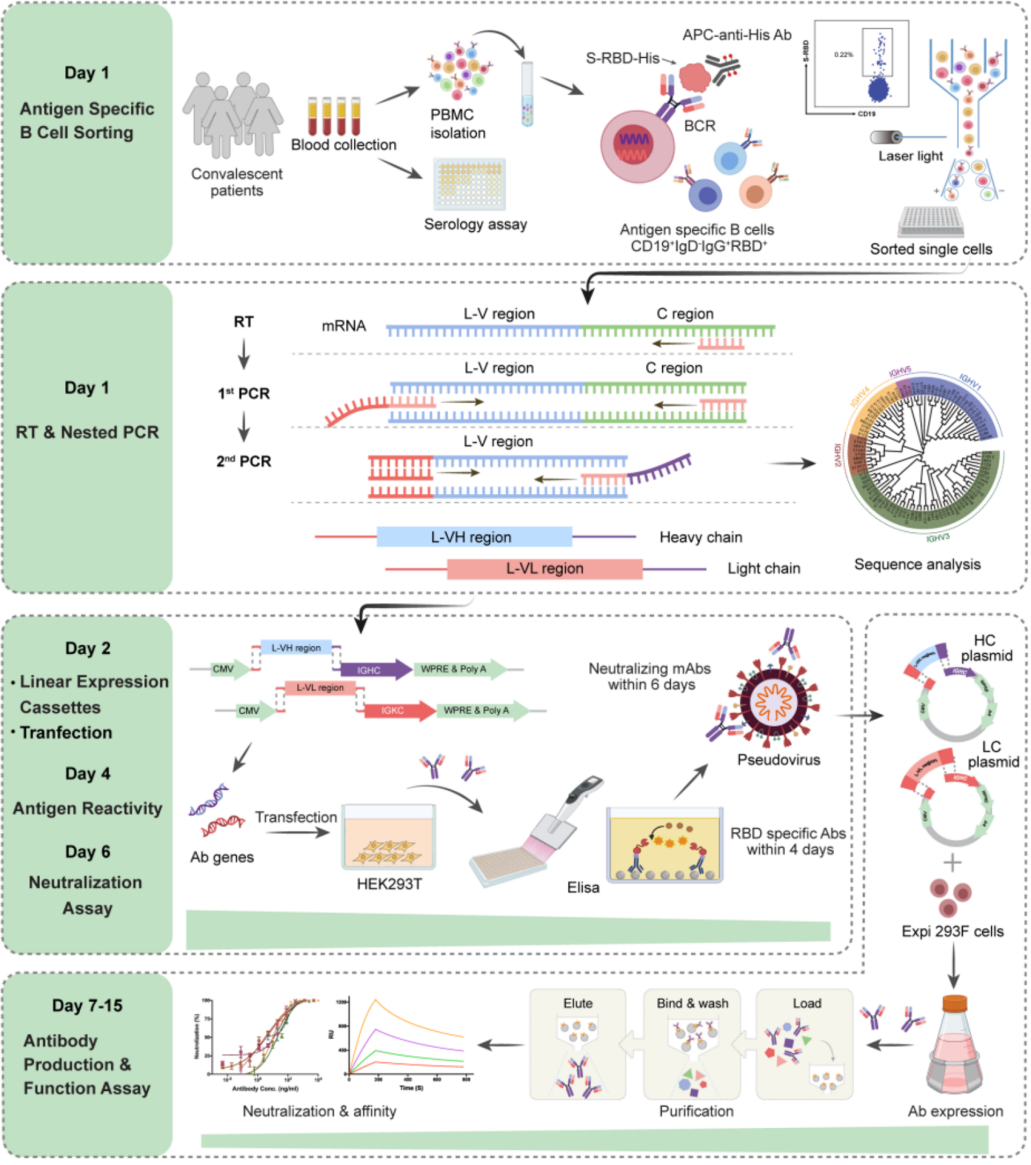
Schematic overview of the rapid and efficient NAbs screening system. Rapid NAbs screening workflows and timelines are shown, representing the multiple processes conducted in order. The PBMC were isolated from collected convalescent patients’ blood, and the RBD-specific memory B cells in the PBMC were sorted as single cells *via* flow cytometry (day 1). Then, the IgG heavy and light chains of mAb genes were amplified by RT-PCR on the same day. 2^nd^ PCR products were cloned into the linear expression cassettes (day 2). HEK293T cells expressed antibodies by transient transfection with equal amounts of paired heavy and light chain linear expression cassettes and culture for 48 hours. The supernatants were used to detect the antigen reactivity of antibodies by ELISA in 384-well plates (day 4). The neutralizing activities of antibodies were measured with pseudovirus bearing SARS-CoV-2 S in 96-well plates (day 6). Plasmids expressing potential neutralizing Abs were transfected into Exi293F cells for the large-scale production of Ab proteins. The cell supernatants of Exi293F cells were collected, and the antibody proteins were purified by protein G beads. Antigen reactivity, neutralizing activity, and binding affinity were further accessed *via* ELISA, competitive ELISA, and surface plasmon resonance (SPR). *The figure was created with Biorender.com*.

We established an optimized screening system for NAbs, using antigen specific memory B cells from the PBMC of patients with infectious diseases (**Figure 1**). At first, the blood samples were collected from COVID-19 convalescent patients. Antigen specific memory B cells (mB cells) in a pooled PBMC from 5-7 patients were detected by labeling with RBD of SARS-CoV-2 and sorted into 96-well plates in a single-cell manner. The variable region of immunoglobulin heavy chains (IGH) and light chains (IGK or IGL) were obtained by RT-PCR and nested PCR, with the optimized primers, at Day 1 (**Supplementary Table 1**). DNA recombinant sites were introduced during the second PCR. Next, linear expression cassettes were assembled by overlapping PCR (25), which contained the essential elements for Ab gene transcription, including the CMV promoter, the antibody variable region, the antibody constant region, and the poly(A)-tail (**Supplementary Table 2**). Then, HEK293T cells were transiently transfected with these linear expression cassettes at Day 2. Supernatants of the transfected cells were evaluated for the S and RBD specific binding activities by enzyme-linked immunosorbent assay (ELISA) at Day 4, and their neutralizing capacities to pseudovirus were tested at Day 6. Subsequently, potent neutralizing Abs were expressed and purified for the functional analyses, including antigen reactivity, viral neutralization, and binding affinity, all of which were completed within the next 9 days. Overall, with this screening system, it only takes 15 days to obtain high potent NAbs against SARS-CoV-2.

### Isolation of Antibody Genes From the Single RBD-Specific Memory B Cells

**Figure 2:**
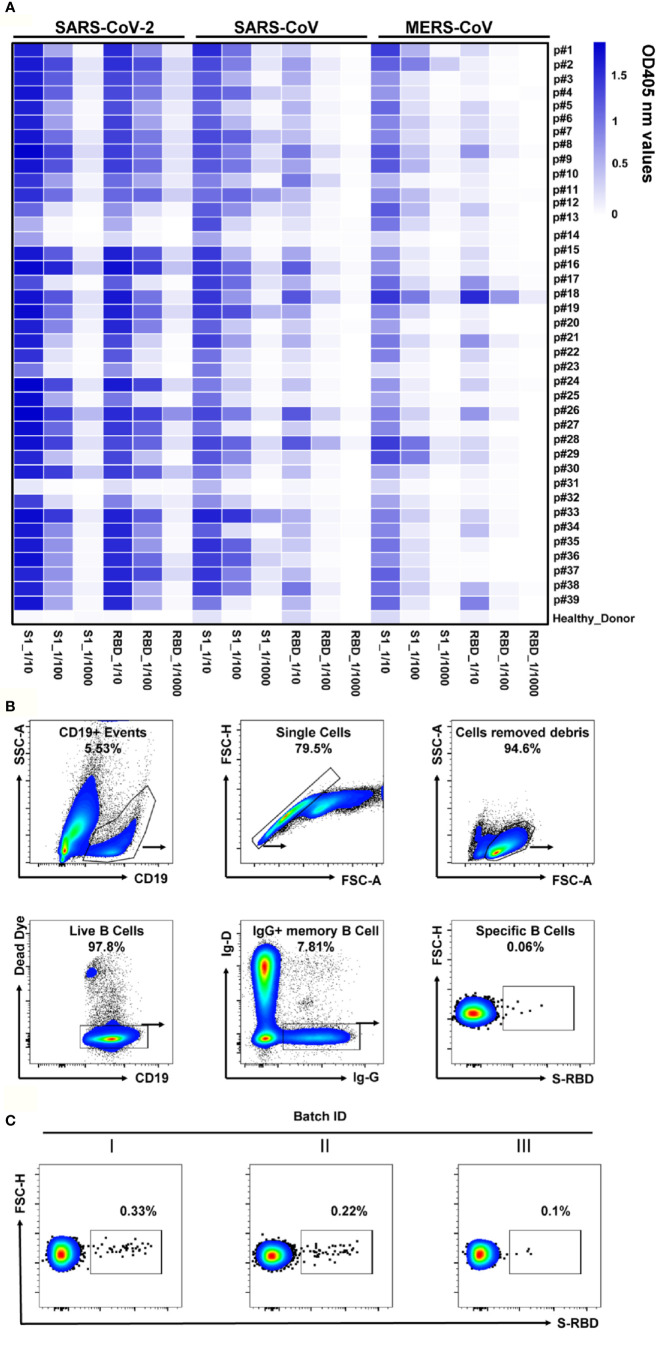
Analyses of plasma response to SARS-CoV-2 and the RBD-specific memory B cells. **(A)** The heatmap depicts the specificity of convalescent patients’ plasma against S1 or RBD of SARS-CoV-2, SARS-CoV, and MERS-CoV, with serial dilutions, measured by ELISA. The plasma of healthy donors was used as a negative control. All results were derived from at least two independent experiments. **(B)** Gating strategy for SARS-CoV-2 RBD-specific IgG^+^ mB cells in the PBMC of COVID-19 convalescent patients. Living CD19^+^IgD^-^IgG^+^ cells were gated, and B cells binding SARS-CoV-2 RBD were selected for single-cell sorting. **(C)** FACS analysis of RBD-specific memory B cells in CD19^+^IgD^-^IgG^+^ mB cells from the PBMC of three batches of convalescent patient samples. Plots show CD19^+^IgD^-^IgG^+^ populations using the gating strategy described in **(B)**.

We collected the blood samples of 39 COVID-19 convalescent patients admitted to Chongqing Medical University affiliated Yongchuan Hospital (**Supplementary Table 3**). These convalescent plasma had been preliminarily screened and confirmed with the positive virus-specific binding and neutralizing capacities, using a magnetic chemiluminescence enzyme immunoassay (MCLIA) and a pseudovirus-based assay (26). Antibody binding to Spike or to the recombinant RBDs of SARS-CoV-2, SARS-CoV or MERS-CoV was quantified using ELISA assay with 10-fold dilution of the patients’ plasma. Among the 39 convalescent plasma samples, 36 showed high reactivity to SARS-CoV-2 S or RBD proteins, while the other three had weak responses (**Figure 2A**). Almost all samples cross-reacted with the S1 protein of SARS-CoV or that of MERS-CoV, with either 10-fold or 100-fold dilutions, while the healthy donor’s plasma did not show a reaction to any of these three coronaviruses (**Figure 2A**). With these findings, we concluded that all samples could be used for RBD-specific Abs isolation to screen NAb candidates.

The RBD is the key domain of SARS-CoV-2 S protein for the interaction with the human cell surface receptor ACE2 (9, 27). Therefore, the recombinant RBD was employed to detect the specific mB cells *via* flow cytometry. We analyzed RBD-specific mB cells by a gating strategy of the Dead Dye^-^CD19^+^IgG^+^IgD^-^RBD^+^cells (**Figure 2B**), the proportion of which was less than 1% in IgD^-^IgG^+^ mB cells (ranging from 0.1% to 0.33%, **Figure 2C**). These RBD-specific mB cells were then sorted into 96-well plates in single cells for Ab gene isolation. Immunoglobulin heavy and light chains were amplified by RT-PCR and nested PCR from the sorted single mB cells (**Supplementary Figure 1A, B** **)**. The amplified products were cloned into linear expression cassettes for producing the full-length IgG antibodies (**Supplementary Figure 1C**). After three rounds of screening, as shown in **Figure 1**, we obtained a total of 497 paired Ab genes from the sorted RBD-specific mB cells (**Supplementary Table 4**).

### Assessment of the Antigen Binding Ability and the Neutralizing Activity of Abs Expressed by Linear Expression Cassettes

**Figure 3:**
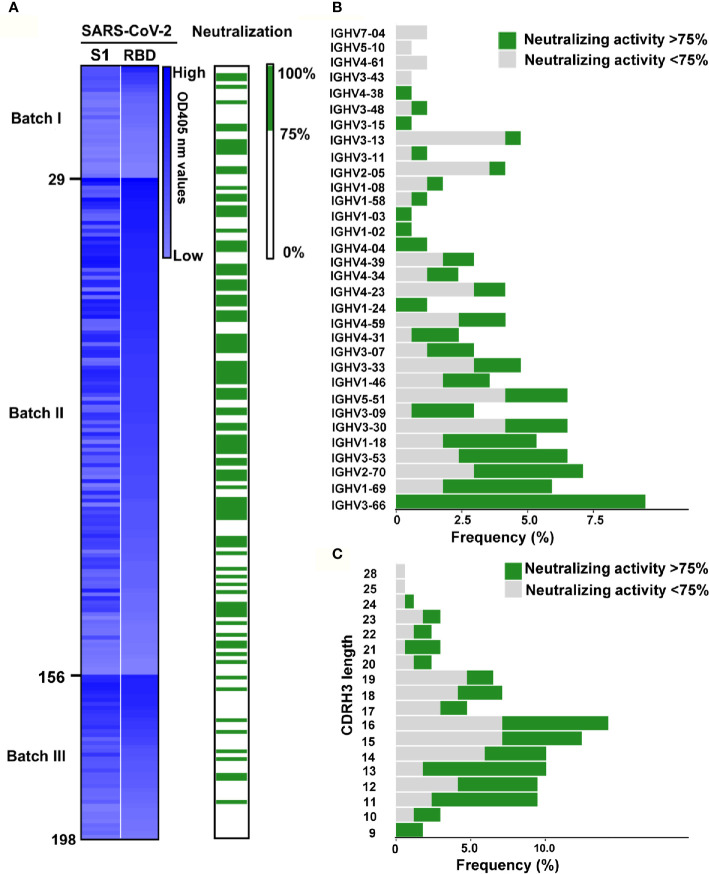
Identification of RBD specific monoclonal antibodies from convalescent COVID-19 patients. **(A)** Screening of SARS-CoV-2 potential neutralizing Abs. The heatmap reveals that the binding ability of 198 Ab supernatants produced by HEK239T cells transfected with linear Ab gene expression cassette. The binding activity of mAbs against SARS-CoV-2 S1 and RBD were tested by ELISA. mAbs were ranked as the binding ability of RBD in each batch, and the brightness of blue represents the binding ability.The OD was measured at 405 nm. The neutralizing activity of mAbs was discriminated according to the neutralizing value. The neutralizing capability was identified by SARS-CoV-2 pseudovirus neutralization assay. The Green columns represent potential neutralizing Abs with the inhibition rate >75%, while white indicate the other Abs. The mAbs were ranked as same as the above. For each evaluated antibody, at least two independent measurements were performed. **(B)** Frequencies of variable region of the heavy chain (VH) gene clusters for potential neutralizing and non-neutralizing antibodies. Clonal sequences groups were collapsed and treated as one sample for calculation of the frequencies. **(C)** Frequency of various the heavy chain complementarity determining region 3 (CDRH3) length of in potential neutralizing and non-neutralizing antibodies.

To identify the specificity of these Abs, Immunoglobulin G (IgG) were expressed in HEK293T cells by transient transfection of the linear expression cassettes carrying paired Ab genes. After two days, the supernatants were measured for IgG1 secretion and the binding capabilities to the recombinant S1 or RBD protein of SARS-CoV-2. In total, we identified 198 RBD specific antibodies from the 497 paired Ab genes (**Figure 3A**). The SARS-CoV-2 pseudovirus neutralization assay was used to assess the neutralizing abilities of these specific antibodies’ supernatants. Interestingly, close to 50% of these mAbs (96/198) could block the pseudovirus infection with over 75% inhibition rate (**Figure 3A**), suggesting that the RBD was an ideal region to screen NAbs against SARS-CoV-2. These results have shown that our screening system can rapidly and efficiently obtain potent NAbs using RBD-specific mB cells.

### Characterization of RBD-Specific Antibody Repertoire

**Figure 4:**
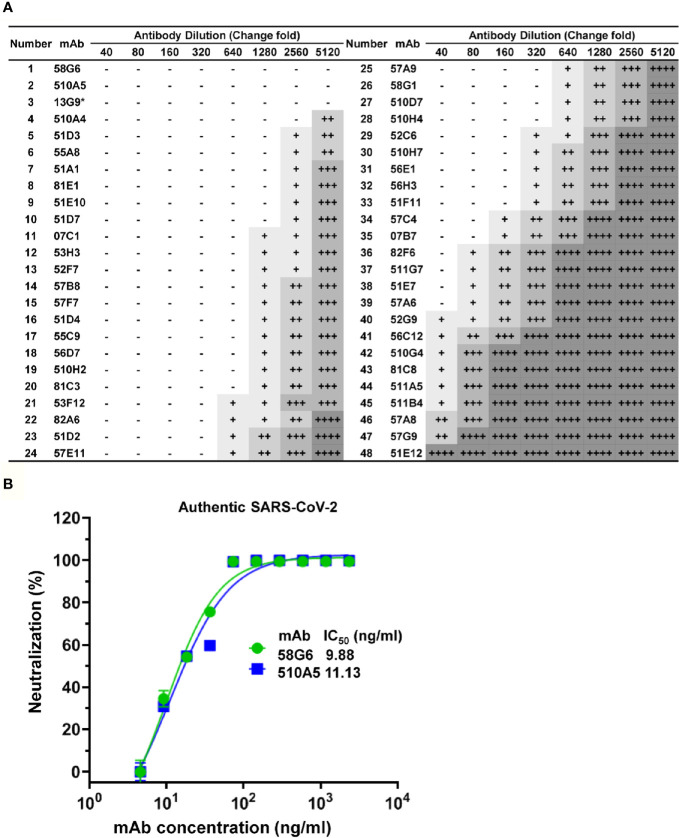
Functional characterization of NAbs against authentic SARS-CoV-2. **(A)** The neutralization activities of mAbs against authentic SARS-CoV-2 virus (nCoV-SH01), analyzed by the Cytopathic effects (CPE) test. Serial dilutions of each mAbs were tested, initial concentration is 0.75 mg/ml. CPE results was summarized, where “-” indicates no cytopathy, “+” indicates <25% cytopathy, “++” indicates 25-50% cytopathy, “+++” indicates 50-75% cytopathy, and “++++” indicates 100% cytopathy. 13G9 was marked with “*” to indicate that it was obtained by the method previously described (28). **(B)** IC_50_ of 58G6 and 510A5 against the authentic SARS-CoV-2 virus, determined in Vero-E6 cells by RT-qPCR. Dashed lines indicated a 50% inhibition rate of viral infection. Data for each NAb were obtained from a representative neutralization experiment, with three replicates. Data are presented as mean ± SEM.

We successfully sequenced 169 of 198 RBD-specific Abs. Among them, 158 (93.5%) were found to have unique sequences, with diverse usage of antibody variable genes (**Supplementary Table 5**). We analyzed the usage of antibody variable-gene segments for variable (V) genes (**Figure 3B**, **Supplementary Figure 2A**). Ninety-six Abs with the neutralizing effect of over 75% against pseudovirus were termed as potential NAbs. Interestingly, we found that all mAbs with heavy chains encoded by IGHV3-66 were potential NAbs (**Figure 3B**), and IGHV3-66 gene could pair with multiple light chain V genes (IGKV1-33, IGKV1-9 and IGLV1-40) (**Supplementary Figure 2C**). Additionally, a large number of mAbs with light chain encoded by IGKV1-39 were potential NAbs (**Supplementary Figure 2A**), and IGKV1-39 gene could pair with a bundle of heavy chains V genes to express RBD specific mAbs (**Supplementary Figure 2C**), which was consistent with previous reports (10, 11). We found that majority of the RBD-specific Abs were transcribed from IGHV1 to IGHV5 for the heavy chain, and IGKV1 to IGKV3 and IGLV1 to IGLV3 for the light chain (**Supplementary Figure 3**). Specifically, close to 50% of the potential NAbs were transcribed from IGHV3 for the heavy chain (**Supplementary Figure 3A**), and IGKV1 for the light chain (**Supplementary Figure 3B**). Of note, the top 2 NAbs (58G6, 510A5) (**Figure 4A**) were transcribed from IGHV1-58 and IGKV1-39, respectively.

The heavy chain complementarity determining region 3 (CDRH3) is the region of an antibody with the highest diversities in amino acid sequence and length. The average length of CDRH3 in the naive human repertoire is round 15 amino acids with a normal distribution (29). We observed that the CDRH3 lengths of the specific mAbs were mainly distributed between 11 to 19 amino acids, while the overall CDRH3 lengths matched the skew distribution (**Figure 3C**). Among them, most of the potential NAbs contained 11-16 amino acids (**Figure 3C**). The mean CDRH3 length in isolated SARS-CoV-2 RBD-specific mB cells differed substantially from those of other viral infections, such as HIV and influenza virus (30, 31). In terms of the CDR3 light chain (CDRL3) lengths, a range of 6 to 13 amino acids were observed, with a similar skew distribution (**Supplementary Figure 2B**).

### Identification of the Potent Neutralizing Antibodies With High Affinity

These 96 potential NAbs were transiently expressed in Expi293F cells. From a total of 96 culture supernatants, we successfully harvested 73 purified mAbs. These purified mAbs were tested for the RBD reactivity by ELISA, and we found that 65 mAbs could bind to SARS-CoV-2 S1 and SARS-CoV-2 RBD (**Supplementary Figure 4A**). Next, the 65 mAbs were assessed their neutralizing ability *in vitro via* competitive ELISA analysis. We found that over 70% of them could block the ACE-2-RBD interaction, suggesting these mAbs might exhibit neutralizing capability. (**Supplementary Figure 4B**).

**Figure 5:**
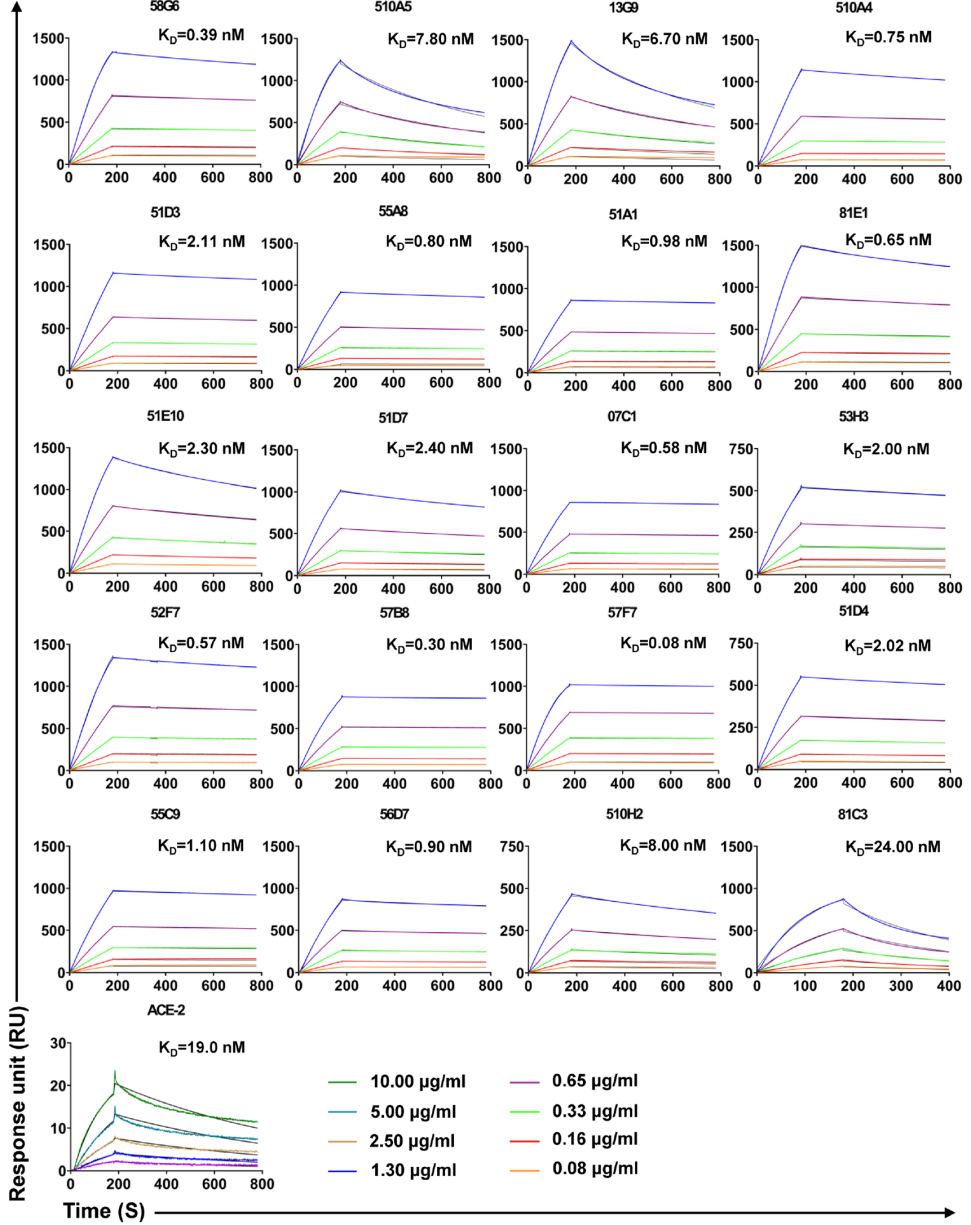
The binding kinetics of the top 20 NAbs with SARS-CoV-2 S-RBD measured by SPR. The purified S-RBD NAbs were captured on the CM5 chip followed by the injection of various concentrations of soluble SARS-CoV-2 S-RBD. In terms of assessment of ACE2 affinity, S-RBD was coated on the CM5 chip. The various concentrations of ACE2 were injected to the coated chip. The color lines represent experimentally derived curves, while the black lines represent fitted curves based on the experimental data. Data are representative of at least 2 independent experiments.

Forty-eight Abs selected from 65 potential NAbs were evaluated for their neutralizing capabilities using the authentic SARS-CoV-2 cytopathic effect (CPE) inhibition assay, and the results were listed according to the order of inhibitory ability (**Figure 4A**). Among them, 20 mAbs were able to completely block the authentic SARS-CoV-2 infection at a diluted concentration of 1.17 µg/ml (640 fold dilution), and the top 2 mAbs (58G6, 510A5) could completely inhibit the authentic SARS-CoV-2 infection below 0.15 µg/ml (5120 fold dilution). Furthermore, half-maximal inhibitory concentration (IC_50_) values of 58G6 and 510A5 were determined by RT-qPCR method using authentic SARS-CoV-2 virus infection. We found that the IC_50_ values of 58G6 and 510A5 were 9.98 and 11.13 ng/ml, respectively (**Figure 4B**). Moreover, we measured the binding affinity of top 20 NAbs to SARS-CoV-2 RBD *via* the surface plasmon resonance (SPR) (**Figure 5**). These mAbs had equilibrium constant (K_D_) values for the binding affinity ranging from 0.08 to 8 nM, except 81C3 (K_D_= 24 nM) (**Table 1**). Among of them, the K_D_ of 58G6 and 510A5 were 0.39 nM and 7.80 nM. Altogether, the most potent neutralizing mAbs (58G6 and 510A5) were obtained and could be used as candidate biologics to prevent or treat SARS-CoV-2 infection using our developed screening system.

**Table 1:** Binding capacity analysis of the top 20 NAbs.

Number	Antibody	Viral protein binding				
		EC_50_-S1 (µg/ml)	EC_50_-S-RBD (µg/ml)	Association (Kka,1/Ms)	Dissociation (Kkd,1/s)	Affnity (K_D_, nM)
**1**	**58G6**	**0.032**	**0.034**	**8.64×10^5^**	**3.32×10^-4^**	**0.39**
**2**	**510A5**	**0.049**	**0.057**	**2.78×10^5^**	**2.17×10^-3^**	**7.80**
**3**	**13G9**	**0.026**	**0.035**	**7.25×10^5^**	**4.87×10^-3^**	**6.70**
**4**	**510A4**	**0.039**	**0.050**	**1.64×10^6^**	**1.24×10^-3^**	**0.75**
**5**	**51D3**	**0.054**	**0.058**	**5.56×10^4^**	**1.17×10^-4^**	**2.11**
**6**	**55A8**	**0.031**	**0.035**	**3.44×10^5^**	**2.75×10^-4^**	**0.80**
**7**	**51A1**	**0.026**	**0.024**	**6.52×10^4^**	**6.36×10^-5^**	**0.98**
**8**	**81E1**	**0.043**	**0.038**	**1.07×10^6^**	**6.99×10^-4^**	**0.65**
**9**	**51E10**	**0.070**	**0.101**	**4.53×10^5^**	**1.05×10^-3^**	**2.30**
**10**	**51D7**	**0.037**	**0.044**	**4.74×10^5^**	**1.15×10^-3^**	**2.40**
**11**	**07C1**	**0.095**	**0.106**	**9.66×10^4^**	**5.56×10^-5^**	**0.58**
**12**	**53H3**	**0.022**	**0.037**	**7.92×10^4^**	**1.58×10^-4^**	**2.00**
**13**	**52F7**	**0.025**	**0.029**	**5.11×10^5^**	**2.90×10^-4^**	**0.57**
**14**	**57B8**	**0.013**	**0.023**	**1.15×10^5^**	**3.45×10^-5^**	**0.30**
**15**	**57F7**	**0.079**	**0.124**	**4.63×10^5^**	**3.80×10^-5^**	**0.08**
**16**	**51D4**	**0.088**	**0.131**	**7.22×10^4^**	**1.46×10^-4^**	**2.02**
**17**	**55C9**	**0.029**	**0.061**	**7.91×10^4^**	**8.76×10^-5^**	**1.10**
**18**	**56D7**	**0.027**	**0.051**	**2.71×10^5^**	**2.44×10^-4^**	**0.90**
**19**	**510H2**	**0.060**	**0.086**	**5.56×10^4^**	**4.46×10^-4^**	**8.00**
**20**	**81C3**	**0.046**	**0.057**	**2.43×10^5^**	**5.82×10^-3^**	**24.00**

## Discussion

Neutralizing antibody-based therapeutic is an ideal medicine for treatment and prevention of the infectious diseases (32). Although NAbs against SASR-CoV-2 could be obtained from convalescent patients, the success rate to discover potent NAbs with therapeutic promise remains unidealized. In this study, we described a strategically optimized screening method to discover potent mAbs from a large number of the antigen specific Abs.

Compared with conventional methods for screening NAbs (11, 20–22), several strategic optimizations of our screening system were discussed below (**Supplementary Figure 6** **)**. Firstly, we utilized the RBD of SARS-CoV-2 as bait to label specific mB cells, which would usually experience affinity maturation and somatic hypermutation (33, 34). SARS-CoV-2 RBD has been proven to be essential for the ACE2-binding during virus entry (8). Although SARS-CoV-2 S could also be applied as bait, it could bring a large number of non-neutralizing antibodies derived from non-RBD regions (13, 18, 19). Secondly, we pooled PBMCs from 5-7 convalescent patients for sorting RBD-specific mB cells. This could reduce duplicated clones to only 6.5% in total, as compared to approximately 20% in other studies (13, 21), and improve the overall screening efficiency. Thirdly, we optimized multiple steps for the single cell BCR cloning and expression (35, 36) (**Supplementary Figure 1A**, **Supplementary Tables 1, 2**). In the 1^st^ PCR step, we designed primers targeting the initial 20 nucleotides at the 5’ end of the signal peptide of Ab genes as the forward primers, which can reduce the loss of BCR clones caused by SNP at the primer binding sites. In the 2^nd^ PCR step, 5’ end of PCR products containing the adaptor primers (AP) could be used for the simultaneous construction of linear gene expression cassettes and plasmids without the extra-modification. Besides, using 3’ end primer from Ab J-region instead of 3’ end primer from Ig constant region in the 2^nd^ PCR, we were able to improve the recombination efficiency of linear cassettes to approaching 100%. Construction of the linear expression cassettes instead of plasmids, which not only lower the cost, but also could drastically reduce the workload and time. Lastly, preliminary evaluation of neutralization activity on, as early as, the sixth day could filter and reduce the number of the Abs to be applied in the subsequent purification and functional characterization steps. Efficiency of every steps were described in **Supplementary Table 4**.

Using this optimized screening system, we efficiently generated a panel of NAbs with relatively great potency. When we analyzed the distribution of gene clusters of BCR repertoire of potential neutralizing and non-neutralizing antibody sequences, a few interesting observations were found. Our results revealed that potential NAbs tended to be distributed in several gene clusters, such as VH3-66 and VH3-53, etc., among which, the VH3-66 cluster exclusively produced NAbs. This result may be helpful in analyzing the preference distribution of genes encoding NAbs in the future. Meanwhile, CDRH3 length is reported as a key factor to value the diversity of RBD specific Abs, due to the changeable amino acid compositions. We found that the CDRH3 lengths of potential NAbs showed a skewed distribution, with an inclined length of 11-16 amino acids.

One additional improvement that may be integrated into our screening system is the single-cell sequencing method. The development of proper algorithms to evaluate neutralization capabilities with the incorporation of heavy chain variable region preferences, for example, IGHV3-66, could help to precisely predict NAbs from repertoires containing thousands of antigen specific mAb (14, 15). This might further provide desired candidates of NAbs with potential therapeutic value, with better time-efficiency and economical preferences.

Based on the screening strategy described above, we successfully identified 20 potent NAbs that can completely block authentic SARS-CoV-2 virus infection at 1.17 μg/ml and have K_D_ values ranging from 0.08 to 8 nM. The top two antibodies (58G6 and 510A5) both generated IC_50_ at around 10 ng/ml, with high binding affinity to SARS-CoV-2 RBD, which were found to be some of the most potent NAbs discovered to date. In conclusion, we have successfully integrated and optimized an optimized screening system for NAbs, which can generate a large number of desired NAbs against SARS-CoV-2 in a total period of 15 days. Our screening system could be expanded to other infectious diseases and serve as a fundamental methodology for discovering NAbs for emerging infectious diseases.

## Data Availability Statement 

The original contributions presented in the study are included in the article/**Supplementary Material**. Further inquiries can be directed to the corresponding authors.

## Ethics Statement

The project “The application of antibody tests patients infected with SARS-CoV-2” was approved by the ethics committee of Chongqing Medical University. The patients/participants provided their written informed consent to participate in this study.

## Author Contributions

AJ and AH conceived and designed the study. KD and FGo offered help on the collection of convalescent patient blood samples. XH performed the fluorescence-activated cell sorting. XH, CHu, LL, and QC performed the single B cell PCR experiments. YWu, RW, FGo, JHu, SM, YL, SS, and YH constructed the linear antibodies gene expression cassettes. FL and JJH were responsible for antibody expression and purification. JW, KW, JHu, SLo, NT, GZ, and QG conducted the pseudovirus neutralization assays, YX, CG, YWu, WX, XC and DQ performed authentic SARS-CoV-2 neutralization assays. SLi, MS, YWu, XH, and AJ analyzed the antibody sequences. SLi, MS, YWu, WW, XH, and JW generated figures and tables, and take responsibility for the integrity and accuracy of data presentation. XH, AJ, and WW wrote the manuscript. All authors contributed to the article and approved the submitted version.

## Funding

This study was supported by Chongqing Medical University fund (X4457) with the donation from Mr. Yuling Feng.

## Conflict of Interest

A patent has been filed for some of the antibodies presented here.

The authors declare that the research was conducted in the absence of any commercial or financial relationships that could be construed as a potential conflict of interest.
