# Supplementary material for: A Rapid and Efficient Screening System for Neutralizing Antibodies and Its Application for SARS-CoV-2
Source: Front Immunol. 2021 Mar 22;12:653189. doi: 10.3389/fimmu.2021.653189 (PMC8019923; doi:10.3389/fimmu.2021.653189)
Supplement: Supplementary file 1 [file DataSheet_1.pdf]

Supplementary Table 1. Primers List of BCR RT-PCR

Primer_Name	V/D/J gene segments	Primer_Sequence(5`>3`)	Usage description
GV_01	IGHV1-18*01	CGGTACCGCGGGCCCGGGAatggactggacctggagcat	AP_G_Leader_Mix
GV_02	IGHV1-2*01	CGGTACCGCGGGCCCGGGAatggactggacctggaggat	As forward primer of heavy chain 1st PCR
GV_03	IGHV1-24*01	CGGTACCGCGGGCCCGGGAatggactgcacctggaggat	
GV_04	IGHV1-38-4*01	CGGTACCGCGGGCCCGGGAatggactggaactggaggat	
GV_05	IGHV1-45*01	CGGTACCGCGGGCCCGGGAatggactggacctggagaat	
GV_06	IGHV1-46*01	CGGTACCGCGGGCCCGGGAatggactggacctggagggt	
GV_07	IGHV1-58*01	CGGTACCGCGGGCCCGGGAatggactggatttggaggat	
GV_08	IGHV1-69*01	CGGTACCGCGGGCCCGGGAatggactggacctggaggtt	
GV_09	IGHV2-26*01	CGGTACCGCGGGCCCGGGAatggacacactttgctacac	
GV_10	IGHV2-5*01	CGGTACCGCGGGCCCGGGAatggacacactttgctccac	
GV_11	IGHV2-70*01	CGGTACCGCGGGCCCGGGAatggacatactttgttccac	
GV_12	IGHV2/OR16-5*01	CGGTACCGCGGGCCCGGGAatggacacgttttgctccac	
GV_13	IGHV3-11*01	CGGTACCGCGGGCCCGGGAatggagtttgggctgagctg	
GV_14	IGHV3-13*01	CGGTACCGCGGGCCCGGGAatggagttggggctgagctg	
GV_15	IGHV3-16*01	CGGTACCGCGGGCCCGGGAatggaatttgggctgagctg	
GV_16	IGHV3-21*01	CGGTACCGCGGGCCCGGGAatggaactggggctccgctg	
GV_17	IGHV3-43*01	CGGTACCGCGGGCCCGGGAatggagtttggactgagctg	
GV_18	IGHV3-48*01	CGGTACCGCGGGCCCGGGAatggagttggggctgtgctg	
GV_19	IGHV3-49*01	CGGTACCGCGGGCCCGGGAatggagtttgggcttagctg	
GV_20	IGHV3-53*01	CGGTACCGCGGGCCCGGGAatggagttttggctgagctg	
GV_21	IGHV3-64*01	CGGTACCGCGGGCCCGGGAatgacggagtttgggctgag	
GV_22	IGHV3-64D*06	CGGTACCGCGGGCCCGGGAatggagttctggctgagctg	
GV_23	IGHV3-7*01	CGGTACCGCGGGCCCGGGAatggaattggggctgagctg	
GV_24	IGHV3-9*01	CGGTACCGCGGGCCCGGGAatggagttgggactgagctg	
GV_25	IGHV4-28*01	CGGTACCGCGGGCCCGGGAatgaaacacctgtggttctt	
GV_26	IGHV4-38-2*02	CGGTACCGCGGGCCCGGGAatgaagcacctgtggttttt	
GV_27	IGHV4-39*01	CGGTACCGCGGGCCCGGGAatgaagcacctgtggttctt	
GV_28	IGHV4-59*01	CGGTACCGCGGGCCCGGGAatgaaacatctgtggttctt	
GV_29	IGHV5-10-1*02	CGGTACCGCGGGCCCGGGAatgcaagtgggggcctctcc	
GV_30	IGHV5-51*01	CGGTACCGCGGGCCCGGGAatggggtcaaccgccatcct	
GV_31	IGHV6-1*01	CGGTACCGCGGGCCCGGGAatgtctgtctccttcctcat	
KV_01	IGKV1/OR2-0*01	CGGTACCGCGGGCCCGGGAatgagggcccccactcagct	AP_K_Leader_Mix
KV_02	IGKV1/OR2-108*01	CGGTACCGCGGGCCCGGGAatggaaatgagggtccccgc	As forward primer of kappa light chain 1st PCR
KV_03	IGKV1-16*01	CGGTACCGCGGGCCCGGGAatggacatgagagtcctcgc	
KV_04	IGKV1-27*01	CGGTACCGCGGGCCCGGGAatggacatgagggtccctgc	
KV_05	IGKV1-5*01	CGGTACCGCGGGCCCGGGAatggacatgagggtccccgc	
KV_06	IGKV1-8*01	CGGTACCGCGGGCCCGGGAatgaggtccccgctcagct	
KV_07	IGKV1D-16*01	CGGTACCGCGGGCCCGGGAatggacatgagggtcctcgc	
KV_08	IGKV1D-43*01	CGGTACCGCGGGCCCGGGAatggacatgagggtgcccgc	
KV_09	IGKV2-24*01	CGGTACCGCGGGCCCGGGAatgaggctccttgctcagct	
KV_10	IGKV2-28*01	CGGTACCGCGGGCCCGGGAatgaggctccctgctcagct	
KV_11	IGKV3/OR2-268*01	CGGTACCGCGGGCCCGGGAatggaagccccagcacagct	
KV_12	IGKV3-15*01	CGGTACCGCGGGCCCGGGAatggaagccccagcgcagct	
KV_13	IGKV3-20*01	CGGTACCGCGGGCCCGGGAatggaaaccccagcgcagct	
KV_14	IGKV3-7*01	CGGTACCGCGGGCCCGGGAatggaagccccagctcagct	
KV_15	IGKV3D-7*01	CGGTACCGCGGGCCCGGGAatggaaccatggaagcccca	
KV_16	IGKV4-1*01	CGGTACCGCGGGCCCGGGAatggtgttgcagacccaggt	
KV_17	IGKV5-2*01	CGGTACCGCGGGCCCGGGAatggggtcccaggttcacct	
KV_18	IGKV6-21*01	CGGTACCGCGGGCCCGGGAatgttgccatcacaactcat	
KV_19	IGKV6D-41*01	CGGTACCGCGGGCCCGGGAatggtgtccccgttgcaatt	
LV_01	IGLV1-40*01	CGGTACCGCGGGCCCGGGAatggcctggtctcctctcct	AP_L_Leader_Mix
LV_02	IGLV1-41*01	CGGTACCGCGGGCCCGGGAatgacctgctcccctctcct	As forward primer of lambda light chain 1st PCR
LV_03	IGLV1-47*02	CGGTACCGCGGGCCCGGGAatggccggcttccctctcct	
LV_04	IGLV10-54*02	CGGTACCGCGGGCCCGGGAatgccctgggctctgctcct	
LV_05	IGLV11-55*01	CGGTACCGCGGGCCCGGGAatggccctgactcctctcct	
LV_06	IGLV2-8*02	CGGTACCGCGGGCCCGGGAatggcctgggctctgctgct	
LV_07	IGLV3-1*01	CGGTACCGCGGGCCCGGGAatggcatggatccctctctt	
LV_08	IGLV3-10*02	CGGTACCGCGGGCCCGGGAatggcctggacccctctcct	
LV_09	IGLV3-19*01	CGGTACCGCGGGCCCGGGAatggcctggacccctctctg	
LV_10	IGLV3-21*01	CGGTACCGCGGGCCCGGGAatggcctggaccgttctcct	
LV_11	IGLV3-25*02	CGGTACCGCGGGCCCGGGAatggcctggatccctctact	
LV_12	IGLV3-27*01	CGGTACCGCGGGCCCGGGAatggcctggatccctctcct	
LV_13	IGLV3-9*02	CGGTACCGCGGGCCCGGGAatggcctggaccgctctcct	
LV_14	IGLV4-3*01	CGGTACCGCGGGCCCGGGAatggcctgggtctccttcta	
LV_15	IGLV4-60*02	CGGTACCGCGGGCCCGGGAatggcctggaccccactcct	
LV_16	IGLV5-39*02	CGGTACCGCGGGCCCGGGAatggcctggactcctctcct	
LV_17	IGLV6-57*02	CGGTACCGCGGGCCCGGGAatggcctgggctccactact	
LV_18	IGLV7-43*01	CGGTACCGCGGGCCCGGGAatggcctggactcctctctt	
LV_19	IGLV8-61*02	CGGTACCGCGGGCCCGGGAatggcctggatgatgcttct	
LV_20	IGLV8/OR8-1*02	CGGTACCGCGGGCCCGGGAatggcctgcatgatgcttct	
LV_21	IGLV9-49*02	CGGTACCGCGGGCCCGGGAatggcctgggctcctctgct	
IGHJ_01	IGHJ1*01	GATGGGCCCTTGGTGGAGGGTGAGGAGACGGTGACCAGGG	IGHJ_region_Primer_Mix
IGHJ_02	IGHJ2*01	GATGGGCCCTTGGTGGAGGGTGAGGAGACAGTGACCAGGG	
IGHJ_03	IGHJ3*01	GATGGGCCCTTGGTGGAGGGTGAAGAGACGGTGACCATTG	
IGHJ_04	IGHJ6*01	GATGGGCCCTTGGTGGAGGGTGAGGAGACGGTGACCGTGG	
IGKJ_01	IGKJ1*01	GATGGTGCAGCCACAGTTCGTTTGATTTCCACCTTGGTCC	IGKJ_region_Primer_Mix
IGKJ_02	IGKJ2*01	GATGGTGCAGCCACAGTTCGTTTGATCTCCAGCTTGGTCC	
IGKJ_03	IGKJ3*01	GATGGTGCAGCCACAGTTCGTTTGATATCCACTTTGGTCC	
IGKJ_04	IGKJ4*01	GATGGTGCAGCCACAGTTCGTTTGATCTCCACCTTGGTCC	
IGKJ_05	IGKJ5*01	GATGGTGCAGCCACAGTTCGTTTAATCTCCAGTCGTGTCC	
IGLJ_01	IGLJ1*01	GGGGCAGCCTTGGGCTGACCTAGGACGGTGACCTTGGTCC	IGLJ_region_Primer_Mix
IGLJ_02	IGLJ2*01	GGGGCAGCCTTGGGCTGACCTAGGACGGTCAGCTTGGTCC	
IGLJ_03	IGLJ4*01	GGGGCAGCCTTGGGCTGACCTAAAATGATCAGCTGGGTTC	
IGLJ_04	IGLJ5*01	GGGGCAGCCTTGGGCTGACCTAGGACGGTCAGCTCGGTCC	
IGLJ_05	IGLJ5*02	GGGGCAGCCTTGGGCTGACCTAGGACGGTCAGCTCCGTCC	
IGLJ_06	IGLJ6*01	GGGGCAGCCTTGGGCTGACCGAGGACGGTCACCTTGGTGC	
IGLJ_07	IGLJ7*01	GGGGCAGCCTTGGGCTGACCGAGGACGGTCAGCTGGGTGC	
IGLJ_08	IGLJ7*02	GGGGCAGCCTTGGGCTGACCGAGGGCGGTCAGCTGGGTGC	
G289-primer	N.D.	TCTTGTCCACCTTGGTGTTGCT	As reverse primer of heavy chain RT & 1st PCR
G97-primer	N.D.	AGTAGTCCTTGACCAGGCAGCCCAG	As reverse primer of heavy chain 2nd PCR
K244-primer	N.D.	GTTTCTCGTAGTCTGCTTTGCTCA	As reverse primer of kappa light chain RT & 1st PCR
K194-primer-01	N.D.	GTGCTGTCCTTGCTGTCCTGCT	As reverse primer of kappa light chain 2nd PCR
K194-primer-02	N.D.	GTGCTGTCCTTGCT**C**TCCTGCT	
L81-primer	N.D.	CACCAGTGTGGCCTTGTTGGCTTG	As reverse primer of lambda light chain RT & 1st PCR
L19-primer-01	N.D.	GGGCGGGAACAGAGTGACC	As reverse primer of lambda light chain 2nd PCR
L19-primer-02	N.D.	GGGCGGGAACAGAGTGAC**A**	
L19-primer-03	N.D.	GGG**T**GGGAACAGAGTGACC	
AP3	N.D.	CGGTACCGCGGGCCCGGGA	As forward primer of 2nd PCR
G20FP	N.D.	CCCTCCACCAAGGGCCCATC	Construction of the linear antibody expression cassettes
K20FP	N.D.	CGAACTGTGGCTGCACCATC	
L20FP	N.D.	GGTCAGCCCAAGGCTGCCCC	
CMV-FP-01	N.D.	AGATATACGCGTTGACATTG	
CMV-RP	N.D.	TCCCGGGCCCGCGGTACCG	
WPRE-RP	N.D.	AGCCCCAGCTGCGCAGATCT	

Supplementary Table 2. The annotation of linear antibody expression cassettes

Linear expression cassettes For IGHC

CMV-FP_Primer<br>CMV promoter<br>CMV-RP_Primer<br>G20_Primer<br>hman IGHG1 Constant region<br>WPRE<br>poly(A) signal<br>WPRE_RP_Primer	[AGATATACGCGTTGACATTG] [ATTATTGACTAGTTATTAATAGTAATCAATTACGGGGTCATTAGTTCATAGCCCATATATGGAGTTCCGCGTTACATAACTTACGGTAAATGGCCCGCCTGGCTGACCGCCCAACGACCCCCGCCCATTGACGTCAATAAT<br>GACGTATGTTCCCATAGTAACGCCAATAGGGACTTTCCATTGACGTCAATGGGTGGAGTATTTACGGTAAACTGCCCACTTGGCAGTACATCAAGTGTATCATATGCCAAGTACGCCCCCTATTGACGTCAATGACGGTAAATGGCCCGCCTGGCATTATGCCC<br>AGTACATGACCTTATGGGACTTTCCTACTTGGCAGTACATCTACGTATTAGTCATCGCTATTACCATGGTGATGCGGTTTTGGCAGTACATCAATGGGCGTGGATAGCGGTTTGACTCACGGGGATTTCCAAGTCTCCACCCCATTGACGTCAATGGGAGTTTG<br>TTTTGGCACCAAAATCAACGGGACTTTCCAAAATGTCGTAACAACTCCGCCCCATTGACGCAAATGGGCGGTAGGCGTGTACGGTGGGAGGTCTATATAAGCAGAGCTCGTTTAGTGAACCGTCAGATCGCCTGGAGACGCCATCCACGCTGTTTTGACCTCCA<br>]TAGAAGACACCGGGACCGATCCAGCCTCCGGACTCTAGACTTCGAATTCTGCAGTCGA [CGGTACCGCGGGCCCGGGA][*2nd-PCR*<br>*product*][CCCTCCACCAAGGGCCCATC] [GGTCTTCCCCCTGGCACCCTCCTCCAAGAGCACCTCTGGGGGCACAGCGGCCCTGGGCTGCCTGGTCAAGGACTACTTCCCCGAACCGGTGACGGTGTCGTGGAACTCAGGCGCCCTGACCAGCGGCGTGCAC<br>ACCTTCCCGGCTGTCCTACAGTCCTCAGGACTCTACTCCCTCAGCAGCGTGGTGACCGTGCCCTCCAGCAGCTTGGGCACCCAGACCTACATCTGCAACGTGAATCACAAGCCCAGCAACACCAAGGTGGACAAGAAAGTTGAGCCCAAATCTTGTGACAAAAC<br>TCACACATGCCCACCGTGCCCAGCACCTGAACTCCTGGGGGGACCGTCAGTCTTCCTCTTCCCCCCAAAACCCAAGGACACCCTCATGATCTCCCGGACCCCTGAGGTCACATGCGTGGTGGTGGACGTGAGCCACGAAGACCCTGAGGTCAAGTTCAACTGGT<br>ACGTGGACGGCGTGGAGGTGCATAATGCCAAGACAAAGCCGCGGGAGGAGCAGTACAACAGCACGTACCGTGTGGTCAGCGTCCTCACCGTCCTGCACCAGGACTGGCTGAATGGCAAGGAGTACAAGTGCAAGGTCTCCAACAAAGCCCTCCCAGCCCCCATC<br>GAGAAAACCATCTCCAAAGCCAAAGGGCAGCCCCGAGAACCACAGGTGTACACCCTGCCCCCATCCCGGGATGAGCTGACCAAGAACCAGGTCAGCCTGACCTGCCTGGTCAAAGGCTTCTATCCCAGCGACATCGCCGTGGAGTGGGAGAGCAATGGGCAGCC<br>GGAGAACAACTACAAGACCACGCCTCCCGTGCTGGACTCCGACGGCTCCTTCTTCCTCTACAGCAAGCTCACCGTGGACAAGAGCAGGTGGCAGCAGGGGAACGTCTTCTCATGCTCCGTGATGCATGAGGCTCTGCACAACCACTACACGCAGAAGAGCCTCT<br>CCCTGTCTCCGGGTAAATGA] **GAATTCGCGGCCGCGAGTTTGATATCT** [CGACAATCAACCTCTGGATTACAAAATTTGTGAAAGATTGACTGGTATTCTTAACTATGTTGCTCCTTTTACGCTATGTGGATACGCTGCTTTAATGCCTTTGTATCATGCTATT<br>GCTTCCCGTATGGCTTTCATTTTCTCCTCCTTGTATAAATCCTGGTTGCTGTCTCTTTATGAGGAGTTGTGGCCCGTTGTCAGGCAACGTGGCGTGGTGTGCACTGTGTTTGCTGACGCAACCCCCACTGGTTGGGGCATTGCCACCACCTGTCAGCTCCTTTC<br>CGGGACTTTCGCTTTCCCCCTCCCTATTGCCACGGCGGAACTCATCGCCGCCTGCCTTGCCCGCTGCTGGACAGGGGCTCGGCTGTTGGGCACTGACAATTCCGTGGTGTTGTCGGGGAAGCTGACGTCCTTTCCATGGCTGCTCGCCTGTGTTGCCACCTGGA<br>TTCTGCGCGGGACGTCCTTCTGCTACGTCCCTTCGGCCCTCAATCCAGCGGACCTTCCTTCCCGCGGCCTGCTGCCGGCTCTGCGGCCTCTTCCGCGTCTTCGCCTTCGCCCTCAGACGAGTCGGATCTCCCTTTGGGCCGCCTCCCCGCCTGG] [AAACGGGG<br>GAGGCTAACTGAAACACGGAAGGAGACAATACCGGAAGGAACCCGCGCTATGACGGCAATAAAAAGACAGAATAAAACGCACGGGTGTTGGGTCGTTTGTTCATAAACGCGGGGTTCGGTCCCAGGGCTGGCACTCTGTCGATACCCCACCGAGACCCCATTGG<br>GGCCAATACGCCCGCGTTTCTTCCTTTTCCCCACCCCACCCCCCAAGTTCGGGTGAAGGCCCAGGGCTCGCAGCCAACGTCGGGGCGGCAGGCCCTGCCATAGC] [AGATCTGCGCAGCTGGGGCT]

Linear expression cassettes For IGKC(kappa)

CMV-FP_Primer<br>CMV promoter<br>CMV-RP_Primer<br>K20_Primer<br>hman IGHG1 Constant region<br>WPRE<br>poly(A) signal<br>WPRE_RP_Primer	[AGATATACGCGTTGACATTG] [ATTATTGACTAGTTATTAATAGTAATCAATTACGGGGTCATTAGTTCATAGCCCATATATGGAGTTCCGCGTTACATAACTTACGGTAAATGGCCCGCCTGGCTGACCGCCCAACGACCCCCGCCCATTGACGTCAATAAT<br>GACGTATGTTCCCATAGTAACGCCAATAGGGACTTTCCATTGACGTCAATGGGTGGAGTATTTACGGTAAACTGCCCACTTGGCAGTACATCAAGTGTATCATATGCCAAGTACGCCCCCTATTGACGTCAATGACGGTAAATGGCCCGCCTGGCATTATGCCC<br>AGTACATGACCTTATGGGACTTTCCTACTTGGCAGTACATCTACGTATTAGTCATCGCTATTACCATGGTGATGCGGTTTTGGCAGTACATCAATGGGCGTGGATAGCGGTTTGACTCACGGGGATTTCCAAGTCTCCACCCCATTGACGTCAATGGGAGTTTG<br>TTTTGGCACCAAAATCAACGGGACTTTCCAAAATGTCGTAACAACTCCGCCCCATTGACGCAAATGGGCGGTAGGCGTGTACGGTGGGAGGTCTATATAAGCAGAGCTCGTTTAGTGAACCGTCAGATCGCCTGGAGACGCCATCCACGCTGTTTTGACCTCCA<br>]TAGAAGACACCGGGACCGATCCAGCCTCCGGACTCTAGACTTCGAATTCTGCAGTCGA [CGGTACCGCGGGCCCGGGA][*2nd-PCR*<br>*product*][CGAACTGTGGCTGCACCATC] [TGTCTTCATCTTCCCGCCATCTGATGAGCAGTTGAAATCTGGAACTGCCTCTGTTGTGTGCCTGCTGAATAACTTCTATCCCAGAGAGGCCAAAGTACAGTGGAAGGTGGATAACGCCCTCCAATCGGGTAAC<br>TCCCAGGAGAGTGTCACAGAGCAGGACAGCAAGGACAGCACCTACAGCCTCAGCAGCACCCTGACGCTGAGCAAAGCAGACTACGAGAAACACAAAGTCTACGCCTGCGAAGTCACCCATCAGGGCCTGAGCTCGCCCGTCACAAAGAGCTTCAACAGGGGAGA<br>GTGTTAG] **GAATTCGCGGCCGCGAGTTTGATATCT** [CGACAATCAACCTCTGGATTACAAAATTTGTGAAAGATTGACTGGTATTCTTAACTATGTTGCTCCTTTTACGCTATGTGGATACGCTGCTTTAATGCCTTTGTATCATGCTATTGCTTCCCGTATGG<br>CTTTCATTTTCTCCTCCTTGTATAAATCCTGGTTGCTGTCTCTTTATGAGGAGTTGTGGCCCGTTGTCAGGCAACGTGGCGTGGTGTGCACTGTGTTTGCTGACGCAACCCCCACTGGTTGGGGCATTGCCACCACCTGTCAGCTCCTTTCCGGGACTTTCGCT<br>TTCCCCCTCCCTATTGCCACGGCGGAACTCATCGCCGCCTGCCTTGCCCGCTGCTGGACAGGGGCTCGGCTGTTGGGCACTGACAATTCCGTGGTGTTGTCGGGGAAGCTGACGTCCTTTCCATGGCTGCTCGCCTGTGTTGCCACCTGGATTCTGCGCGGGAC<br>GTCCTTCTGCTACGTCCCTTCGGCCCTCAATCCAGCGGACCTTCCTTCCCGCGGCCTGCTGCCGGCTCTGCGGCCTCTTCCGCGTCTTCGCCTTCGCCCTCAGACGAGTCGGATCTCCCTTTGGGCCGCCTCCCCGCCTGG] [AAACGGGGGAGGCTAACTGAA<br>ACACGGAAGGAGACAATACCGGAAGGAACCCGCGCTATGACGGCAATAAAAAGACAGAATAAAACGCACGGGTGTTGGGTCGTTTGTTCATAAACGCGGGGTTCGGTCCCAGGGCTGGCACTCTGTCGATACCCCACCGAGACCCCATTGGGGCCAATACGCCC<br>GCGTTTCTTCCTTTTCCCCACCCCACCCCCCAAGTTCGGGTGAAGGCCCAGGGCTCGCAGCCAACGTCGGGGCGGCAGGCCCTGCCATAGC] [AGATCTGCGCAGCTGGGGCT]

Linear expression cassettes For IGLC (lambda)

CMV-FP_Primer<br>CMV promoter<br>CMV-RP_Primer<br>L20_Primer<br>hman IGHG1 Constant region<br>WPRE<br>poly(A) signal<br>WPRE_RP_Primer	[AGATATACGCGTTGACATTG] [ATTATTGACTAGTTATTAATAGTAATCAATTACGGGGTCATTAGTTCATAGCCCATATATGGAGTTCCGCGTTACATAACTTACGGTAAATGGCCCGCCTGGCTGACCGCCCAACGACCCCCGCCCATTGACGTCAATAAT<br>GACGTATGTTCCCATAGTAACGCCAATAGGGACTTTCCATTGACGTCAATGGGTGGAGTATTTACGGTAAACTGCCCACTTGGCAGTACATCAAGTGTATCATATGCCAAGTACGCCCCCTATTGACGTCAATGACGGTAAATGGCCCGCCTGGCATTATGCCC<br>AGTACATGACCTTATGGGACTTTCCTACTTGGCAGTACATCTACGTATTAGTCATCGCTATTACCATGGTGATGCGGTTTTGGCAGTACATCAATGGGCGTGGATAGCGGTTTGACTCACGGGGATTTCCAAGTCTCCACCCCATTGACGTCAATGGGAGTTTG<br>TTTTGGCACCAAAATCAACGGGACTTTCCAAAATGTCGTAACAACTCCGCCCCATTGACGCAAATGGGCGGTAGGCGTGTACGGTGGGAGGTCTATATAAGCAGAGCTCGTTTAGTGAACCGTCAGATCGCCTGGAGACGCCATCCACGCTGTTTTGACCTCCA<br>]TAGAAGACACCGGGACCGATCCAGCCTCCGGACTCTAGACTTCGAATTCTGCAGTCGA [CGGTACCGCGGGCCCGGGA][*2nd-PCR*<br>*product*][GGTCAGCCCAAGGCTGCCCC] [CTCGGTCACTCTGTTCCCGCCCTCCTCTGAGGAGCTTCAAGCCAACAAGGCCACACTGGTGTGTCTCATAAGTGACTTCTACCCGGGAGCCGTGACAGTGGCCTGGAAGGCAGATAGCAGCCCCGTCAAGGCG<br>GGAGTGGAGACCACCACACCCTCCAAACAAAGCAACAACAAGTACGCGGCCAGCAGCTATCTGAGCCTGACGCCTGAGCAGTGGAAGTCCCACAGAAGCTACAGCTGCCAGGTCACGCATGAAGGGAGCACCGTGGAGAAGACAGTGGCCCCTACAGAATGTTC<br>ATAG] **GAATTCGCGGCCGCGAGTTTGATATCT** [CGACAATCAACCTCTGGATTACAAAATTTGTGAAAGATTGACTGGTATTCTTAACTATGTTGCTCCTTTTACGCTATGTGGATACGCTGCTTTAATGCCTTTGTATCATGCTATTGCTTCCCGTATGGCTT<br>TCATTTTCTCCTCCTTGTATAAATCCTGGTTGCTGTCTCTTTATGAGGAGTTGTGGCCCGTTGTCAGGCAACGTGGCGTGGTGTGCACTGTGTTTGCTGACGCAACCCCCACTGGTTGGGGCATTGCCACCACCTGTCAGCTCCTTTCCGGGACTTTCGCTTTC<br>CCCCTCCCTATTGCCACGGCGGAACTCATCGCCGCCTGCCTTGCCCGCTGCTGGACAGGGGCTCGGCTGTTGGGCACTGACAATTCCGTGGTGTTGTCGGGGAAGCTGACGTCCTTTCCATGGCTGCTCGCCTGTGTTGCCACCTGGATTCTGCGCGGGACGTC<br>CTTCTGCTACGTCCCTTCGGCCCTCAATCCAGCGGACCTTCCTTCCCGCGGCCTGCTGCCGGCTCTGCGGCCTCTTCCGCGTCTTCGCCTTCGCCCTCAGACGAGTCGGATCTCCCTTTGGGCCGCCTCCCCGCCTGG] [AAACGGGGGAGGCTAACTGAAACA<br>CGGAAGGAGACAATACCGGAAGGAACCCGCGCTATGACGGCAATAAAAAGACAGAATAAAACGCACGGGTGTTGGGTCGTTTGTTCATAAACGCGGGGTTCGGTCCCAGGGCTGGCACTCTGTCGATACCCCACCGAGACCCCATTGGGGCCAATACGCCCGCG<br>TTTCTTCCTTTTCCCCACCCCACCCCCCAAGTTCGGGTGAAGGCCCAGGGCTCGCAGCCAACGTCGGGGCGGCAGGCCCTGCCATAGC] [AGATCTGCGCAGCTGGGGCT]

Supplementary Table 3. Patient information

Subject	Age (years)	Sex	Subjsct type	Clinical classification (mild:0, severe:1)	City where infected	Date of admission	Date of discharge	Date of blood sample collection	Volume of collected blood sample
P#1	46	F	SARS-CoV-2 convalescence patient	0	Chongqing	2/3/2020	2/28/2020	3/16/2020	30ml
P#2	50	M	SARS-CoV-2 convalescence patient	0	Chongqing	2/2/2020	2/28/2020	3/16/2020	30ml
P#3	54	M	SARS-CoV-2 convalescence patient	1	Chongqing	1/29/2020	2/18/2020	3/16/2020	30ml
P#4	47	M	SARS-CoV-2 convalescence patient	1	Chongqing	1/27/2020	2/23/2020	3/16/2020	30ml
P#5	48	F	SARS-CoV-2 convalescence patient	0	Chongqing	2/16/2020	2/28/2020	3/16/2020	30ml
P#6	31	M	SARS-CoV-2 convalescence patient	0	Chongqing	1/30/2020	2/10/2020	3/16/2020	30ml
P#7	45	F	SARS-CoV-2 convalescence patient	0	Chongqing	2/1/2020	2/15/2020	3/16/2020	30ml
P#8	51	F	SARS-CoV-2 convalescence patient	0	Chongqing	1/31/2020	2/17/2020	3/16/2020	50ml
P#9	48	M	SARS-CoV-2 convalescence patient	1	Chongqing	1/30/2020	2/18/2020	4/1/2020	50ml
P#10	31	M	SARS-CoV-2 convalescence patient	0	Chongqing	1/29/2020	2/18/2020	4/1/2020	50ml
P#11	54	M	SARS-CoV-2 convalescence patient	0	Chongqing	2/2/2020	2/18/2020	4/1/2020	30ml
P#12	47	F	SARS-CoV-2 convalescence patient	0	Chongqing	1/31/2020	2/10/2020	4/1/2020	10ml
P#13	38	M	SARS-CoV-2 convalescence patient	0	Chongqing	2/10/2020	2/19/2020	4/1/2020	10ml
P#14	33	F	SARS-CoV-2 convalescence patient	0	Chongqing	2/10/2020	3/9/2020	4/1/2020	10ml
P#15	44	M	SARS-CoV-2 convalescence patient	0	Chongqing	2/5/2020	2/14/2020	4/1/2020	50ml
P#16	60	F	SARS-CoV-2 convalescence patient	0	Chongqing	2/11/2020	3/5/2020	4/1/2020	30ml
P#17	48	M	SARS-CoV-2 convalescence patient	0	Chongqing	2/12/2020	3/6/2020	4/1/2020	30ml
P#18	53	F	SARS-CoV-2 convalescence patient	0	Chongqing	2/16/2020	2/28/2020	4/1/2020	10ml
P#19	57	M	SARS-CoV-2 convalescence patient	0	Chongqing	1/29/2020	2/14/2020	4/1/2020	10ml
P#20	44	M	SARS-CoV-2 convalescence patient	0	Chongqing	2/6/2020	2/29/2020	4/1/2020	10ml
P#21	44	F	SARS-CoV-2 convalescence patient	0	Chongqing	2/13/2020	3/11/2020	4/1/2020	30ml
P#22	20	F	SARS-CoV-2 convalescence patient	0	Chongqing	2/15/2020	2/25/2020	4/1/2020	30ml
P#23	37	M	SARS-CoV-2 convalescence patient	0	Chongqing	2/2/2020	3/9/2020	4/1/2020	50ml
P#24	44	F	SARS-CoV-2 convalescence patient	0	Chongqing	2/3/2020	2/28/2020	4/1/2020	50ml
P#25	41	M	SARS-CoV-2 convalescence patient	0	Chongqing	1/31/2020	2/16/2020	4/1/2020	14ml
P#26	66	M	SARS-CoV-2 convalescence patient	0	Chongqing	2/3/2020	2/16/2020	4/1/2020	10ml
P#27	67	F	SARS-CoV-2 convalescence patient	0	Chongqing	2/4/2020	2/16/2020	4/1/2020	10ml
P#28	50	M	SARS-CoV-2 convalescence patient	1	Chongqing	2/1/2020	2/29/2020	4/1/2020	30ml
P#29	50	F	SARS-CoV-2 convalescence patient	0	Chongqing	1/28/2020	2/29/2020	4/1/2020	50ml
P#30	49	F	SARS-CoV-2 convalescence patient	0	Chongqing	2/8/2020	3/6/2020	4/1/2020	30ml
P#31	56	M	SARS-CoV-2 convalescence patient	0	Chongqing	2/1/2020	2/9/2020	4/1/2020	30ml
P#32	55	M	SARS-CoV-2 convalescence patient	0	Chongqing	2/14/2020	2/21/2020	4/1/2020	50ml
P#33	45	F	SARS-CoV-2 convalescence patient	0	Chongqing	2/1/2020	2/15/2020	4/1/2020	30ml
P#34	47	F	SARS-CoV-2 convalescence patient	0	Chongqing	2/6/2020	2/17/2020	4/1/2020	10ml
P#35	34	M	SARS-CoV-2 convalescence patient	0	Chongqing	1/28/2020	2/7/2020	4/1/2020	10ml
P#36	47	M	SARS-CoV-2 convalescence patient	1	Chongqing	1/27/2020	2/23/2020	4/1/2020	50ml
P#37	56	M	SARS-CoV-2 convalescence patient	0	Chongqing	1/30/2020	2/7/2020	4/1/2020	40ml
P#38	63	F	SARS-CoV-2 convalescence patient	0	Chongqing	2/1/2020	2/25/2020	4/1/2020	10ml
P#39	37	M	SARS-CoV-2 convalescence patient	0	Chongqing	1/27/2020	2/14/2020	4/1/2020	10ml

Supplementary Table 4. Three batches of S-RBD specific B memory cell sorting and efficiency evaluation

Batch	Sample count	Subject	Volume of blood used for sorting(ml)	Total volume of blood(ml)	Number of sorted single B cells	Number of Paired mAbs after BCR RT-PCR	Number of Specific mAbs (note-01)	Number of Potential Neutralizing mAbs (note-01)	**Efficiency Evaluation** Percent of paired mABs gene in sorted&cloned single cells	Percent of speicifc mAbs in paired mABs gene	Percent of the potential neutralizing mAbs in the specific mABs
0428	1	p#10	10	70	192	72	29	12	38%	40%	41%
	2	p#11	10								
	3	p#29	10								
	4	p#30	10								
	5	p#32	10								
	6	p#36	10								
	7	p#37	10								
0505	1	p#8	10	60	1056	324	127	75	31%	39%	59%
	2	p#16	15								
	3	p#17	15								
	4	p#26	10								
	5	p#27	10								
0528	1	p#01	10	75	432	101	42	9	23%	42%	21%
	2	p#03	10								
	3	p#04	10								
	4	p#06	10								
	5	p#09	10								
	6	p#22	10								
	7	p#25	5								
	8	p#28	5								
	9	p#31	5								
				sum=205	sum=1680	sum=497	sum=198	sum=96	mean=31%	mean=40%	mean=41%

Note-01: the linear expression cassettes were transfected into 293T cells for expressing Ab proteins. The supernatants of cell cultures were collected 48hs later. the specific binding ability were assessed by ELISA. the neutralizaiton capability were assessed by pseudovirus assay,and the mAbs, of which neutralizing effect were over 75%, were termed as Neutralizing mAbs.

Supplementary Table 5. Gene family analysis of monoclonal antibodies

Number	Ab_ID	Heavy chain				Light chain		
		V-GENE and allele	D-GENE and allele	J-GENE and allele	CDR3-IMGT(aa)	V-GENE and allele	J-GENE and allele	CDR3-IMGT(aa)
1	81E8	IGHV2-70*15 F	IGHD6-13*01 F	IGHJ4*02 F	ARVQVAAAGSPYDY	IGKV1-39*01 F	IGKJ1*01 F	QQTYSTPRT
2	81D2	IGHV3-53*01 F	IGHD3-3*01 F	IGHJ4*02 F	ARFRYGDYPDY	IGKV1-33*01 F	IGKJ3*01 F	QQYDNLPSFT
3	81A2	IGHV4-34*01 F	IGHD2-2*02 F	IGHJ6*02 F	GVPYCSSSSCYRYYGMDV	IGKV1-9*01 F	IGKJ5*01 F	QQLNSYPQAT
4	81C8	IGHV3-33*01 F	IGHD3-3*01 F	IGHJ4*02 F	ARDGVDFGMVTLFDY	IGKV1-39*01 F	IGKJ1*01 F	QQSYNTPPWT
5	81A1	IGHV4-59*01 F	IGHD6-19*01 F	IGHJ3*02 F	ARDQGYSSGWNDAFDI	IGLV2-23*01 F	IGLJ3*02 F	CSYAGSRFWV
6	81A11	IGHV1-69*02 F	IGHD1-7*01 F	IGHJ2*01 F	AREAGTTDWYFDL	IGLV1-40*01 F	IGLJ2*01 F	QSYDSSLSVVV
7	81C3	IGHV4-39*01 F	IGHD3-3*01 F	IGHJ4*02 F	ARHPRFSWRGNDSGYFDY	IGLV2-14*01 F	IGLJ1*01 F	SSFTSSSTPYV
8	81C7	IGHV2-5*02 F	IGHD3-10*01 F	IGHJ4*02 F	AHSMVRGVLFGADFDY	IGLV1-36*01 F	IGLJ3*02 F	AAWDDSLNGPV
9	81D5	IGHV5-51*01 F	IGHD1-26*01 F	IGHJ3*02 F	ARRWDGVGFDI	IGLV3-19*01 F	IGLJ1*01 F	NSRDSSINHYV
10	81F2	IGHV4-34*01 F	IGHD1-14*01 ORF	IGHJ5*02 F	ARGWTVPPLWVLNWFDP	IGKV2-28*01 F	IGKJ4*01 F	MQALQTPRT
11	81G8	IGHV3-7*05 F	IGHD4-23*01 ORF	IGHJ4*02 F	ARSPHYYGGFDY	IGLV6-57*02 F	IGLJ3*02 F	QSYHNSVWV
12	81E1	IGHV1-24*01 F	IGHD3-10*01 F	IGHJ4*02 F	AITSVARGLRGYFDT	IGLV1-47*01 F	IGLJ2*01 F	AAWDDSLSRVV
13	81E10	IGHV1-24*01 F	IGHD3-10*01 F	IGHJ4*02 F	AITSLARGLKGYFDS	IGLV1-47*01 F	IGLJ2*01 F	AAWDDSLSGVI
14	81A6	IGHV3-9*01 F	IGHD2-2*01 F	IGHJ6*02 F	AKDIGCSSTSCSYYHYYGMDV	IGKV2-28*01 F	IGKJ2*01 F	MQALQTPT
15	81B2	IGHV3-66*02 F	IGHD6-19*01 F	IGHJ4*02 F	ARDGRAVAGTD	IGKV1-33*01 F	IGKJ1*01 F	HQYDNLPRT
16	81B7	IGHV1-18*04 F	IGHD3-10*01 F	IGHJ4*02 F	ARDYGWFGELSTEGQFDY	IGKV3-11*01 F	IGKJ3*01 F	QQRRDFT
17	82A6	IGHV4-39*01 F	IGHD6-19*01 F	IGHJ4*02 F	ARFITDGYSSGSDS	IGKV1-39*01 F	IGKJ4*01 F	QQSYSTPRLT
18	82C4	IGHV1-58*01 F	IGHD1-26*01 F	IGHJ6*02 F	AADRMRIVGGKGYYYGMDV	IGKV1-5*03 F	IGKJ2*01 F	QQYNSYSPGDT
19	82G8	IGHV4-59*08 F	IGHD2-15*01 F	IGHJ5*02 F	ARRSMGYCSGGNCYSGFDP	IGKV1-39*01 F	IGKJ4*01 F	QQSYSTPLT
20	82B6	IGHV4-61*04 F	IGHD3-16*01 F	IGHJ5*02 F	AMTYYDYIWGRVDPQFDP	IGKV2-30*01 F	IGKJ1*01 F	CMQGTHWPPT
21	82B7	IGHV1-18*04 F	IGHD3-10*01 F	IGHJ4*02 F	ARDYGWFGELSTEGQFDY	IGKV3-20*01 F	IGKJ1*01 F	HQYASSPRT
22	82C6	IGHV4-39*01 F	IGHD6-19*01 F	IGHJ4*02 F	ARFITDGYSSGSDS	IGKV2-30*02 [F]	IGKJ2*01 F	MQGTHWPMYT
23	82F6	IGHV3-30*03 F	IGHD2-15*01 F	IGHJ4*02 F	AKQASPYCSGGSCYSGNFDY	IGKV1-33*01 F	IGKJ4*01 F	QQHDNVPLT
24	82A5	IGHV1-18*04 F	IGHD3-9*01 F	IGHJ4*02 F	ARVQWLRLDY	IGKV1-12*01 F	IGKJ4*01 F	QQAKGFPLT
25	82C12	IGHV2-5*02 F	IGHD3-3*01 F	IGHJ4*02 F	AHRPAGFWSAHFDY	IGKV1-39*01 F	IGKJ1*01 F	QQSYSTPAWT
26	51D3	IGHV3-66*01 F	IGHD1-26*01 F	IGHJ6*02 F	AREGLLVGPTGRGLGMDV	IGKV1-33*01 F	IGKJ2*01 F	QQYADLPYT
27	51D4	IGHV2-70*15 F	IGHD3-10*01 F	IGHJ4*02 F	ARMVVRGVMLDY	IGKV1-39*01 F	IGKJ2*03 (F)	QQSYSPPHS
28	51A3	IGHV1-18*04 F	IGHD3-10*01 F	IGHJ4*02 F	ARDLAWFGELSESPIEY	IGKV3-11*01 F	IGKJ5*01 F	QQRGNSIT
29	51D2	IGHV2-5*01 F	IGHD3-9*01 F	IGHJ3*01 F	AHRLAPDYDFLTGYYNGDDAFDV	IGKV1-39*01 F	IGKJ4*01 F	QQSYNTLALS
30	51A1	IGHV3-66*01 F	IGHD5-12*01 F	IGHJ3*02 F	ARDLNIAGGFDI	IGKV1-9*01 F	IGKJ5*01 F	QHLNIDPIT
31	51E12	IGHV3-7*01 F	IGHD2-2*01 F	IGHJ4*02 F	ARLMYYYGNFDY	IGKV1-8*01 F	IGKJ2*01 F	QQYYGYPT
32	51D12	IGHV5-10-1*03 F	IGHD2-2*01 F	IGHJ6*02 F	ARLPFCDSASCGRAHYYYGMDV	IGKV3-15*01 F	IGKJ2*01 F	QQYNNWPET
33	51F11	IGHV1-69*04 F	IGHD5-18*01 F	IGHJ4*02 F	ATGRYTYGYGYYFDY	IGKV3-20*01 F	IGKJ2*02 (F)	QQYGSSRT
34	51E10	IGHV1-18*04 F	IGHD6-13*01 F	IGHJ2*01 F	ARARQLVLNWYFDL	IGKV4-1*01 F	IGKJ4*01 F	QQYYITPQLT
35	51E7	IGHV3-13*01 F	IGHD3-10*01 F	IGHJ2*01 F	ARVGYYGSGSYPLYWYFDL	IGKV1-39*01 F	IGKJ1*01 F	QQSYSAPPWT
36	51D7	IGHV5-51*01 F	IGHD6-19*01 F	IGHJ3*02 F	ATRTGWTNDAFDI	IGKV1-39*01 F	IGKJ2*02 (F)	QQSYSTPCT
37	51F5	IGHV2-70*13 F	IGHD1-7*01 F	IGHJ4*02 F	ALGRAGTMDY	IGKV1-39*01 F	IGKJ2*01 F	QQSYSPPYT
38	51E3	IGHV3-9*01 F	IGHD6-19*01 F	IGHJ6*02 F	AKDMVAGPHYYGMDV	IGLV1-44*01 F	IGLJ3*02 F	AAWDDSLNGWV
39	51B1	IGHV5-51*01 F	IGHD3-10*01 F	IGHJ6*02 F	ARHPSNFYDSGGDYYAMDV	IGLV2-23*02 F	IGLJ3*02 F	CSYAGSSSWV
40	52C1	IGHV3-66*01 F	IGHD3-10*01 F	IGHJ4*02 F	ARLASDGSGSYLDYFDY	IGLV1-40*01 F	IGLJ3*02 F	QSYDSSLSGSWV
41	52G1	IGHV3-9*01 F	IGHD6-19*01 F	IGHJ4*02 F	AKDLSSGWDLFDY	IGLV3-21*04 F	IGLJ2*01 F	QVWDTSGDPLV
42	52C6	IGHV1-69*01 F	IGHD1-26*01 F	IGHJ6*02 F	ATDGGGGSYYYAHYYYGMDV	IGLV8-61*01 F	IGLJ2*01 F	VLYMGSGIVV
43	52E7	IGHV5-51*01 F	IGHD4-17*01 F	IGHJ4*02 F	VRSDGDYVIGHDY	IGLV1-44*01 F	IGLJ3*02 F	ATWDDSLNGRV
44	52B6	IGHV3-48*03 F	IGHD4-17*01 F	IGHJ4*02 F	ARGRDDYGDYRGGDFDY	IGKV3-11*01 F	IGKJ3*01 F	QQRSNWPPIT
45	52F7	IGHV3-9*01 F	IGHD2-2*01 F	IGHJ6*02 F	AKDIGVMVPGVTPYGMDV	IGKV2D-30*01 F	IGKJ2*02 (F)	MQGTHWPPGT
46	52B8	IGHV1-69*01 F	IGHD3-10*01 F	IGHJ3*02 F	ARGPAYVSGTYYWNAFDI	IGKV3-15*01 F	IGKJ2*01 F	QHYNNWPLYT
47	52E9	IGHV3-30*04 F	IGHD6-13*01 F	IGHJ3*02 F	ARDPPPGNMGSMAQHLVLLVVFDI	IGKV1-39*01 F	IGKJ4*01 F	QQSYTTLALT
48	52G9	IGHV3-66*01 F	IGHD6-25*01 F	IGHJ6*02 F	ARDPMRPGMDV	IGKV1-33*01 F	IGKJ1*01 F	QQYDNLPRT
49	53H3	IGHV3-66*01 F	IGHD4-17*01 F	IGHJ3*02 F	ARYYGPQGRAFDI	IGKV1-33*01 F	IGKJ3*01 F	QQYDNLPLT
50	53C5	IGHV1-69*09 F	IGHD5-18*01 F	IGHJ4*02 F	ARGRYTYGTEGYFDN	IGKV1-5*01 F	IGKJ2*01 F	QQYNNLYT
51	53G1	IGHV3-33*03 F	IGHD6-19*01 F	IGHJ4*02 F	AKGGFGYASGWYYLDY	IGKV1-33*01 F	IGKJ5*01 F	QQYGNLPPIA
52	53B2	IGHV3-13*01 F	IGHD3-22*01 F	IGHJ4*02 F	ARAYYDTSGYYNYFDH	IGKV3-15*01 F	IGKJ2*01 F	QHYNNWPLYT
53	53H5	IGHV3-53*01 F	IGHD3-10*01 F	IGHJ5*02 F	ARGYGSGSYGWFDP	IGKV3-15*01 F	IGKJ2*01 F	QHYNNWPLYT
54	53H6	IGHV3-53*04 F	IGHD1-26*01 F	IGHJ6*02 F	AREGLGMDV	IGKV3-15*01 F	IGKJ2*01 F	QHYNNWPLYT
55	53F9	IGHV3-53*04 F	IGHD1-26*01 F	IGHJ4*02 F	AREGLVGTTLTFDY	IGKV2D-30*01 F	IGKJ2*02 (F)	MQGTHWPPGT
56	53G6	IGHV2-70*15 F	IGHD1-26*01 F	IGHJ5*02 F	ARFLVGGFKAWFDP	IGLV1-51*01 F	IGLJ3*02 F	GTWDTSLNVWV
57	53C10	IGHV3-43*02 F	IGHD1-20*01 F	IGHJ4*02 F	ARESPKLTGYFDY	IGLV3-21*02 F	IGLJ1*01 F	QVWDSSSDPYV
58	53E11	IGHV1-46*01 F	IGHD5-12*01 F	IGHJ4*02 F	ARDQAFIVATLGPDY	IGLV8-61*01 F	IGLJ3*02 F	VLYMGSGIWV
59	53E12	IGHV3-53*01 F	IGHD5-12*01 F	IGHJ5*02 F	AKMLWLRGWFDP	IGLV6-57*03 F	IGLJ3*02 F	QSYDTSNHWV
60	53F12	IGHV3-66*01 F	IGHD4-23*01 ORF	IGHJ6*02 F	ARDAVGSYYYGMEV	IGKV3-15*01 F	IGKJ2*01 F	QHYNNWPLYT
61	54H2	IGHV5-51*01 F	IGHD6-19*01 F	IGHJ4*02 F	ARQESGWSFDY	IGKV1-39*01 F	IGKJ1*01 F	QQSYSTPRT
62	54F10	IGHV3-53*04 F	IGHD5-24*01 ORF	IGHJ6*02 F	ARDLEERGAMDV	IGKV3-20*01 F	IGKJ1*01 F	QQYGSSLWT
63	54G7	IGHV3-66*01 F	IGHD3-16*02 F	IGHJ5*02 F	ARAGWLRGRFDP	IGLV3-21*02 F	IGLJ3*02 F	QVWDSTTDLPHWV
64	55G3	IGHV2-70*15 F	IGHD1-14*01 ORF	IGHJ3*02 F	ARTRLGITAFDI	IGKV1-39*01 F	IGKJ1*01 F	QQSYTTPRT
65	55A8	IGHV1-69*04 F	IGHD4-17*01 F	IGHJ4*02 F	ARGTEYGDYDVSHD	IGKV1-5*03 F	IGKJ2*01 F	QQYNSYSHT
66	55C9	IGHV3-53*04 F	IGHD2-15*01 F	IGHJ4*02 F	AREGLVGTALAFDY	IGKV1-39*01 F	IGKJ2*01 F	QQSYSTPPYT
67	55G9	IGHV3-13*01 F	IGHD3-22*01 F	IGHJ2*01 F	ARVGYDSSGYYWYLDL	IGKV1-39*01 F	IGKJ3*01 F	QQSYTTPLFT
68	55G11	IGHV3-30*03 F	IGHD2-15*01 F	IGHJ4*02 F	GKGAGPYCGGGSCYPTKVDY	IGKV1-33*01 F	IGKJ1*01 F	QQYDNLLWT
69	56C3	IGHV3-33*01 F	IGHD3-10*01 F	IGHJ4*02 F	AKDGSGSYYNSGALDY	IGKV1-33*01 F	IGKJ2*01 F	QQYDNLPPYT
70	56H3	IGHV3-66*01 F	IGHD4-17*01 F	IGHJ4*02 F	ARDYGDYYFDY	IGKV3-20*01 F	IGKJ1*01 F	QQYGSSPRT
71	56B4	IGHV1-18*01 F	IGHD2-2*01 F	IGHJ4*02 F	ATDDPDIVLVPAAMSLDY	IGKV1-39*01 F	IGKJ4*01 F	QQSYNTLALS
72	56D7	IGHV3-66*01 F	N.D.	IGHJ6*02 F	ARDLDYYGMDV	IGKV1-9*01 F	IGKJ5*01 F	QQLNSYPPIT
73	56H7	IGHV4-59*01 F	IGHD3-22*01 F	IGHJ6*02 F	ATDYYDSSGYRYGMDV	IGKV1D-12*01 F	IGKJ4*01 F	QQANSFPLT
74	56A8	IGHV1-69*01 F	IGHD1-26*01 F	IGHJ4*02 F	ASFGSLWDLRDY	IGKV3-20*01 F	IGKJ4*01 F	QQYGTSRT
75	56E8	IGHV3-23*01 F	IGHD2-15*01 F	IGHJ4*02 F	AKDVGSRLIYDVFDY	IGKV1-39*01 F	IGKJ2*03 (F)	QQSYSTPPYS
76	56A10	IGHV2-70*15 F	IGHD4-23*01 ORF	IGHJ4*02 F	TRTATVVKDY	IGKV1-39*01 F	IGKJ1*01 F	QQSYSTPRT
77	56B11	IGHV2-70*15 F	IGHD6-6*01 F	IGHJ4*02 F	ARMIPIPALDY	IGKV1-39*01 F	IGKJ2*01 F	QQSYSTPHT
78	56B12	IGHV2-70*15 F	IGHD6-13*01 F	IGHJ4*02 F	AREEAAGTKLDY	IGKV1-39*01 F	IGKJ1*01 F	QQSYSTPRT
79	56C12	IGHV3-30*03 F	IGHD2-15*01 F	IGHJ5*02 F	AKDPTSLYCSGGSCYNNWFDP	IGKV1-39*01 F	IGKJ1*01 F	QQTYSTPRT
80	56E1	IGHV3-30*04 F	IGHD3-16*01 F	IGHJ3*02 F	AGGGVLVTSDPDAFDI	IGLV1-44*01 F	IGLJ3*02 F	AAWDDSLNGWV
81	56A3	IGHV3-30*03 F	IGHD2-15*01 F	IGHJ4*02 F	AKRGGTYCSGGICYGGYFDY	IGLV1-40*01 F	IGLJ3*02 F	QSYDSSLSDWGV
82	56F3	IGHV1-46*01 F	IGHD3-10*01 F	IGHJ4*02 F	ARDYSRITMIRGAGDY	IGLV1-51*01 F	IGLJ3*02 F	GTWDSSLSAQV
83	56H9	IGHV3-53*04 F	IGHD6-13*01 F	IGHJ5*02 F	ARGPYPSSSWA	IGLV2-14*01 F	IGLJ1*01 F	SSFTSSSTPYV
84	56H11	IGHV4-4*07 F	IGHD5-12*01 F	IGHJ4*02 F	AGEQHIVTTIIDY	IGLV1-44*01 F	IGLJ3*02 F	ATWDDSLNGRV
85	57B2	IGHV3-30*04 F	IGHD3-16*01 F	IGHJ3*02 F	AGGGVLVTSDPDAFDI	IGKV1-33*01 F	IGKJ3*01 F	QQSDNVPVT
86	57C4	IGHV3-15*01 F	IGHD4-17*01 F	IGHJ5*01 F	STTNDYGDYSANY	IGKV1-39*01 F	IGKJ4*01 F	QQSYSTPLT
87	57E7	IGHV5-51*01 F	IGHD3-22*01 F	IGHJ4*02 F	ARIEYYNDSSGYYQF	IGKV3-20*01 F	IGKJ1*01 F	QQYGSSWT
88	57H7	IGHV1-8*02 F	IGHD3-10*01 F	IGHJ5*02 F	ARGLWFGDLTRTKYNWFDP	IGKV1D-13*01 F	IGKJ4*01 F	QQFNNFLLT
89	57A8	IGHV3-23*01 F	IGHD1-26*01 F	IGHJ4*02 F	AKGQRGSPDFFDY	IGKV1-5*03 F	IGKJ4*01 F	QQYNSYSPLT
90	57B8	IGHV3-53*01 F	IGHD2-21*02 F	IGHJ4*02 F	ARDLVTWGLDY	IGKV1-9*01 F	IGKJ5*01 F	QLLNTDPIT
91	57A9	IGHV1-3*04 (F)	IGHD1-26*01 F	IGHJ4*02 F	ARAGWELNY	IGKV4-1*01 F	IGKJ1*01 F	QQYYSFWA
92	57G9	IGHV2-70*15 F	IGHD6-6*01 F	IGHJ4*02 F	ARITPHLVYDY	IGKV1-39*01 F	IGKJ2*02 (F)	QQSYSIPRT
93	57A6	IGHV5-51*01 F	IGHD6-19*01 F	IGHJ4*02 F	ARQESGWSFDY	IGLV3-19*01 F	IGLJ3*02 F	NSRDSSGNHWV
94	57F7	IGHV4-34*01 F	IGHD6-6*01 F	IGHJ6*02 F	ARDDSSSSGVGTGMDV	IGLV1-44*01 F	IGLJ3*02 F	AVWDDSLNGWV
95	57F8	IGHV2-5*02 F	IGHD3-10*01 F	IGHJ5*02 F	AHTTWYYYGSGWFDP	IGLV1-44*01 F	IGLJ2*01 F	AAWDDSLNGHV
96	57A11	IGHV1-18*01 F	IGHD3-10*01 F	IGHJ5*02 F	ARVQEFWLDP	IGLV3-21*04 F	IGLJ3*02 F	QVWDSSSDHPL
97	57E11	IGHV1-8*02 F	IGHD3-10*01 F	IGHJ5*02 F	ARGLWFGDLTRTKYNWFDP	IGLV1-44*01 F	IGLJ3*02 F	AAWDDSLNGWV
98	58G1	IGHV3-53*01 F	IGHD4-17*01 F	IGHJ6*02 F	ARDLENGGLDV	IGKV1D-12*01 F	IGKJ4*01 F	QQTNSFPT
99	58D2	IGHV3-11*01 F	IGHD3-10*01 F	IGHJ2*01 F	ASPLLSHNYGSGSYYNVYWYFEL	IGKV3-11*01 F	IGKJ2*02 (F)	QQLGT
100	58A4	IGHV4-59*01 F	IGHD3-9*01 F	IGHJ5*02 F	ARTLGAYYDILTGFRTPGGWFAP	IGKV3-20*01 F	IGKJ1*01 F	QQYGSSPWT
101	58B5	IGHV3-23*01 F	IGHD3-10*01 F	IGHJ4*02 F	AKEPSFGLWFGELSG	IGKV3-20*01 F	IGKJ2*01 F	QQYGSSLPYT
102	58G6	IGHV1-58*01 F	IGHD2-2*01 F	IGHJ3*02 F	AAPNCNSTTCHDGFDI	IGKV3-20*01 F	IGKJ1*01 F	QQYDNSPWT
103	58G9	IGHV3-13*04 F	IGHD3-9*01 F	IGHJ3*02 F	ARGSLRGGILSGYAFDI	IGKV1-39*01 F	IGKJ1*01 F	QQSYSSPPWT
104	58E2	IGHV4-39*07 F	IGHD3-10*01 F	IGHJ3*02 F	ARYYGPGTYYDAFDI	IGLV2-14*01 F	IGLJ3*02 F	SSYTSSSTWV
105	58A3	IGHV3-30*04 F	IGHD3-10*01 F	IGHJ4*02 F	ARDWVHYGSGSYPPDY	IGLV2-18*02 F	IGLJ3*02 F	SSYTSSSTWV

106	58H3	IGHV5-51*01 F	IGHD3-10*01 F	IGHJ4*02 F	ARHPVLRGNIDY	IGLV1-40*01 F	IGLJ3*02 F	QSYDSSLRGSV
107	510H2	IGHV3-66*01 F	IGHD5-12*01 F	IGHJ4*02 F	ARDKWEGTFDY	IGKV1-9*01 F	IGKJ4*01 F	QQLNSYPRMT
108	510A4	IGHV2-70*15 F	IGHD6-13*01 F	IGHJ4*02 F	ARVQVAAAGSPYDY	IGKV2-28*01 F	IGKJ1*01 F	MQALQMGT
109	510G4	IGHV4-31*03 F	IGHD4-23*01 ORF	IGHJ4*02 F	ARDYGGNSNYFHY	IGKV1-33*01 F	IGKJ4*01 F	QQYDTLPLT
110	510H4	IGHV3-66*01 F	IGHD5-18*01 F	IGHJ6*02 F	AETGWDGMDV	IGKV3-11*01 F	IGKJ1*01 F	QQRSNWPGT
111	510A5	IGHV3-9*01 F	IGHD3-9*01 F	IGHJ4*02 F	AKDRGYEILTPASFDY	IGKV1-39*01 F	IGKJ2*01 F	QQSYSTPPYT
112	510F6	IGHV2-5*02 F	IGHD5-18*01 F	IGHJ4*02 F	AHSLPSKYSYSYGSFDY	IGKV1-39*01 F	IGKJ2*01 F	QQSYSAPYT
113	510D7	IGHV1-69*04 F	IGHD5-18*01 F	IGHJ4*02 F	ATGRYTYGYGYYFDY	IGKV3-20*01 F	IGKJ2*02 (F)	QQYGSSRT
114	510E10	IGHV3-23*01 F	IGHD3-10*01 F	IGHJ5*02 F	AKGELLWFGELLENWFDP	IGKV1-12*01 F	IGKJ1*01 F	QQADSFPWT
115	510H10	IGHV2-70*15 F	IGHD6-13*01 F	IGHJ4*02 F	ARIQRGIAADY	IGKV1-39*01 F	IGKJ2*02 (F)	QQSYSTPRT
116	510B1	IGHV5-51*01 F	IGHD3-10*01 F	IGHJ3*02 F	ARLPPHFGSGSYYGNAFDI	IGLV1-47*01 F	IGLJ3*02 F	ATWDDSLTGPV
117	510G2	IGHV7-4-1*02 F	IGHD3-10*01 F	IGHJ6*02 F	ASTVGRGSGTYYYGNYYSYSMDV	IGLV2-14*01 F	IGLJ2*01 F	TSYTGSSTSVV
118	510H7	IGHV4-59*08 F	IGHD6-13*01 F	IGHJ5*02 F	ARHCPWQQLVSNWFDP	IGKV1D-13*01 F	IGKJ4*01 F	QQFNNFLLT
119	510H6	IGHV1-46*01 F	IGHD3-3*01 F	IGHJ6*02 F	ARTGFLIPSKGGGMDV	IGLV2-14*01 F	IGLJ2*01 F	SSYTSSSSLQI
120	510C4	IGHV3-48*03 F	IGHD1-26*01 F	IGHJ4*02 F	ARDPGEWESLDLDY	IGLV1-40*01 F	IGLJ3*02 F	QSYDSSLSGNWV
121	511A1	IGHV4-31*03 F	IGHD6-13*01 F	IGHJ6*02 F	AREKIRSIAAAGTVYYYGMDV	IGKV3-15*01 F	IGKJ1*01 F	QQYNNWPPWT
122	511B4	IGHV4-59*08 F	IGHD3-22*01 F	IGHJ5*01 F	ASTYWDSSGYYYGVDY	IGKV1D-12*01 F	IGKJ4*01 F	QQANSFRLT
123	511E7	IGHV5-51*01 F	IGHD3-10*01 F	IGHJ4*02 F	ALAVGRGIPTSYFDY	IGKV1-33*01 F	IGKJ3*01 F	QQYHNLPIT
124	511G7	IGHV3-33*01 F	IGHD4-17*01 F	IGHJ4*02 F	AKGGNYGDYLRGFDY	IGKV1-33*01 F	IGKJ4*01 F	QQYHNVPPA
125	511E9	IGHV1-18*01 F	IGHD3-10*01 F	IGHJ4*02 F	AREGAGLIIAYDY	IGKV6-21*01 F	IGKJ2*01 F	HQSSSLPYT
126	511D11	IGHV1-18*01 F	IGHD2-15*01 F	IGHJ6*02 F	AVLDYCSGGSSSSGYYNYGMDV	IGKV3-20*01 F	IGKJ2*01 F	QQYGRSPYT
127	511H11	IGHV3-13*04 F	IGHD6-19*01 F	IGHJ6*03 F	VRGDHSSGWYGTYYYYMDV	IGKV1-39*01 F	IGKJ1*01 F	QQSYSSPPWT
128	511D12	IGHV3-66*01 F	IGHD5-12*01 F	IGHJ3*02 F	ARDLDIAGAFDI	IGKV1-9*01 F	IGKJ5*01 F	QLLNSFPIT
129	511A5	IGHV4-31*06 F	IGHD3-22*01 F	IGHJ2*01 F	ARIYRGTMVVVFSDLHWYFDL	IGLV3-21*04 F	IGLJ1*01 F	QVWDSSADHYV
130	511E5	IGHV1-2*02 F	IGHD3-16*01 F	IGHJ2*01 F	ARDSLFSRVDWYFDL	IGLV1-40*01 F	IGLJ2*01 F	NSRDSSGNTVV
131	511G5	IGHV1-46*01 F	IGHD6-6*01 F	IGHJ4*02 F	ARDGALYSNSPTEFDY	IGLV1-47*01 F	IGLJ3*02 F	TTWDASRGGWV
132	511B7	IGHV1-46*01 F	IGHD2-2*01 F	IGHJ4*02 F	ARGGLVPAVMPALDY	IGLV1-47*01 F	IGLJ3*02 F	AAWDDSLSGPV
133	511H7	IGHV3-23*01 F	IGHD3-22*01 F	IGHJ6*02 F	ARGLQYYYDTSGYYKDSYYYGVDV	IGLV1-47*01 F	IGLJ3*02 F	AAWDDSLSGPV
134	511B11	IGHV3-7*03 F	IGHD3-10*01 F	IGHJ4*02 F	AGLFWYGGYFDY	IGLV1-40*01 F	IGLJ1*01 F	QSYDRSLSVLYV
135	59B11	IGHV3-13*04 F	IGHD3-3*01 F	IGHJ6*03 F	ARGTQDRVELMVGSPYYYYYMDV	IGKV1-39*01 F	IGKJ2*01 F	QQSYITTMYT
136	59D6	IGHV4-34*01 F	IGHD3-22*01 F	IGHJ4*02 F	ARHRRDYITMIVRPTRLWAFDY	IGLV1-40*01 F	IGLJ2*01 F	QSYDSALVV
137	59A1	IGHV3-66*01 F	IGHD1-26*01 F	IGHJ4*02 F	ARELGPVGGTDQ	IGKV1-39*01 F	IGKJ2*01 F	QQSHSTPYT
138	59A2	IGHV3-66*01 F	IGHD2-15*01 F	IGHJ4*02 F	ARDLPLHGDYFDY	IGKV1-33*01 F	IGKJ3*01 F	QQSDNVPVT
139	59H6	IGHV3-30*04 F	IGHD2-15*01 F	IGHJ4*02 F	ARETLGGYCNGGSCYDAGYFDY	IGKV3-20*01 F	IGKJ1*01 F	QQYGSSPWT
140	59G12	IGHV3-33*01 F	IGHD3-3*01 F	IGHJ4*02 F	ARDGVDFGMVTLFDY	IGKV1-39*01 F	IGKJ1*01 F	QQSYNTPPWT
141	8B1E6	IGHV4-4*02 F	IGHD3-16*01 F	IGHJ4*02 F	ARVQGLIDY	IGKV1-NL1*01 F	IGKJ2*02 (F)	QQYYSTPPRT
142	8B1B1	IGHV5-51*01 F	IGHD6-13*01 F	IGHJ6*03 F	ASQAAAGYYYYMDV	IGKV1-39*01 F	IGKJ1*01 F	QQSYNILWT
143	8B1H12	IGHV3-23*01 F	IGHD4-17*01 F	IGHJ5*02 F	AKQTDYGVGWFDP	IGKV1-33*01 F	IGKJ4*01 F	QQYDNFSLT
144	8B1E11	IGHV1-69*06 F	IGHD3-10*01 F	IGHJ4*02 F	AVLPLHSSYNWYYFDY	IGKV3-11*01 F	IGKJ2*01 F	QQRSNWPPGMYT
145	8B2F2	IGHV2-5*02 F	IGHD6-13*01 F	IGHJ5*02 F	AHQRHSNSWYVSAWFDP	IGLV1-40*01 F	IGLJ1*01 F	QSYDSSLSGNFV
146	8B5A6	IGHV3-33*01 F	IGHD6-13*01 F	IGHJ4*02 F	AKGGWYSSKWYYFDY	IGKV2-28*01 F	IGKJ1*01 F	MQALQMGT
147	8B2D5	IGHV3-13*01 F	IGHD4-17*01 F	IGHJ3*02 F	ARGSDTVTTAFDI	IGKV1-39*01 F	IGKJ4*01 F	QQSYTTPGLT
148	8B2H8	IGHV3-53*04 F	IGHD3-10*01 F	IGHJ4*02 F	AREAPNSRGSGTNFDY	IGKV1-39*01 F	IGKJ2*01 F	QQSYSTPPYT
149	8B2D11	IGHV3-33*01 F	IGHD5-18*01 F	IGHJ4*02 F	AKNGYSYAYPRQYFDY	IGKV1-33*01 F	IGKJ2*02 (F)	QHYDNLKVT
150	8B2E4	IGHV4-31*03 F	IGHD3-10*01 F	IGHJ4*02 F	ARVSRYTMVRGVIFDY	IGLV2-23*02 F	IGLJ2*01 F	CLYAGHSTYVV
151	8B2G6	IGHV4-38-2*02 F	IGHD2-21*02 F	IGHJ4*02 F	ARVGVATILGVDY	IGLV6-57*03 F	IGLJ3*02 F	QSYDSSWV
152	8B2E12	IGHV3-30*03 F	IGHD6-19*01 F	IGHJ6*02 F	AKGGGYISAWSTRYYAMDV	IGLV2-14*01 F	IGLJ3*02 F	SSYTSSSTWV
153	8B3E9	IGHV3-23*01 F	IGHD6-13*01 F	IGHJ4*02 F	AESSSLTGNFNY	IGLV3-21*03 F	IGLJ3*02 F	QVWDTAWV
154	8B5A1	IGHV3-13*01 F	IGHD5-18*01 F	IGHJ4*02 F	ARGFDTTTGFYFDY	IGKV1-NL1*01 F	IGKJ1*01 F	QQDYNFPWT
155	8B5B6	IGHV1-69*04 F	IGHD3-10*01 F	IGHJ6*03 F	ARTEYSYDSGSSRAYSMDV	IGKV1-39*01 F	IGKJ1*01 F	QQTHSTPRT
156	8B5E5	IGHV7-4-1*02 F	IGHD6-13*01 F	IGHJ5*02 F	ARVGPSSSWPS	IGKV1-39*01 F	IGKJ1*01 F	QQSYSTPRT
157	8B5C1	IGHV3-33*01 F	IGHD6-13*01 F	IGHJ4*02 F	AKGGWYSSKWYYFDY	IGKV2-28*01 F	IGKJ1*01 F	MQALQMGT
158	8B5F7	IGHV3-7*01 F	IGHD3-3*01 F	IGHJ4*02 F	ARDLGVLWFGDPY	IGKV1-NL1*01 F	IGKJ1*01 F	QQYYSAPRT
159	8B4G4	IGHV2-5*02 F	IGHD3-9*01 F	IGHJ4*02 F	AHSPDHRYFDVLTGYFNSERFYFDY	IGLV1-51*01 F	IGLJ2*01 F	GTWDSSLSAGV
160	8B4A7	IGHV4-39*07 F	IGHD6-6*01 F	IGHJ6*02 F	ARIPRHLGQDHYYYVMDV	IGLV2-23*01 F	IGLJ1*01 F	CSYAGIFV
161	8B4D9	IGHV3-30*03 F	IGHD5-18*01 F	IGHJ4*02 F	AKAAGGGYSYIYWGGDY	IGLV6-57*03 F	IGLJ3*02 F	QSYDSSNLWV
162	8B4E1	IGHV4-61*01 F	IGHD2-2*01 F	IGHJ6*02 F	AREYFVSLPAAQTLYYYGIDV	IGKV3-15*01 F	IGKJ1*01 F	QQYKNWPPWT
163	8B4H2	IGHV1-8*02 F	IGHD3-10*01 F	IGHJ5*02 F	ARGLWFGDLTRTKYNWFDP	IGKV3-20*01 F	IGKJ4*01 F	HQYDSSPLT
164	8B4A3	IGHV1-46*01 F	IGHD3-10*01 F	IGHJ6*02 F	ARDPPSGNYDNIDEWTRSENHYNYGMDA	IGKV3-11*01 F	IGKJ4*01 F	QQRSNWPYLT
165	8B4C5	IGHV3-11*06 F	IGHD6-19*01 F	IGHJ4*02 F	ARDGSAVAGPMSYFDY	IGKV1-NL1*01 F	IGKJ1*01 F	QQYYSIPRT
166	8B4E5	IGHV2-70*15 F	IGHD6-19*01 F	IGHJ4*02 F	AREVAGAVHLDY	IGKV1-39*01 F	IGKJ1*01 F	QQSFSTPRT
167	8B4B6	IGHV3-7*03 F	N.D.	IGHJ4*02 F	ARDLGVLWFGDLLF	IGKV1-NL1*01 F	IGKJ1*01 F	QQYYSDPPRT
168	8B4E11	IGHV4-59*01 F	IGHD3-10*01 F	IGHJ4*02 F	ARGGYYYGPPRDFDY	IGKV3-20*01 F	IGKJ2*01 F	QHYGSSPQYT
169	13G9	IGHV1-58*01 F	IGHD2-2*01 F	IGHJ3*02 F	AAPYCSSTSCRDGFDI	IGKV3-20*01 F	IGKJ1*01 F	QQYGRSPWT

Supplementary Table 6. BCR RT-PCR Reaction mixture and PCR Program setup

Section 01. Single Cell BCR RT-PCR Procedure

1. Preparation of PCR Reaction Mixture

(1) Preparation of RT

a. Preparation of RT_Mix_A (5μl/tube)

Seq.	Component	Amount (μl)
1	Water	2.6
2	2.5 mM dNTPs	2
3	BCR_RT_Primer_Mix	0.4

b. Preparation of RT_Mix_B (5μl/tube)

Seq.	Component	Amount (μl)
4	Water	2.25
5	5×PrimeScript II Buffer	2
6	200 U/μl PrimeScript II Reverse Transcriptase	0.5
7	40,000 U/ml RNase Inhibitor, Murine (NEB)	0.25

(2) Preparation of 1st and 2nd PCR (10μl/tube)

1st_PCR_Mix_N and 2nd_PCR_Mix_N use the same reaction condition except the primers.

Seq.	Component	Amount (μl)
1	2×PrimeSTAR GC Buffer (Takara)	5
2	nuclease-free water	2.75
3	2.5 mM dNTP	0.8
4	10 μM Forward Primer	0.2
5	10 μM Reverse Primer	0.2
6	2.5 U/μl PrimeSTAR HS DNA polymerase	0.05
7	Template	0

(3) Usage of primers

a. For 1st_PCR_Mix

	For 1st_PCR_Mix_Gamma	For 1st_PCR_Mix_Kappa	For 1st_PCR_Mix_Lambda
Forward Primer	AP_G_leader Mix	AP_K_leader Mix	AP_L_leader Mix
Reverse Primer	G289_primer(10μM)	K244_Primer(10μM)	L81_Primer(10μM)

b. For 2nd_PCR_Mix

	For 2nd_PCR_Mix_Gamma	For 2nd_PCR_Mix_Kappa	For 2nd_PCR_Mix_Lambda
Forward Primer	10 μM AP_Primer	10 μM AP_Primer	10 μM AP_Primer
Reverse Primer	IGHJ_region_Primer Mix	10 μM K194_Primer Mix	10 μM L19_Primer Mix

2. Operation Procedure

(1) Take out the sorted 96-well plate at -80. Add 5μl RT_Mix_A to each well, and rinse the well to promote cell RNA dissolution.
(2) Incubate the plate at 65° for 5min and put it on ice immediately.
(3) Add 5μl of RT_Mix_B to each well of the plate, mix and centrifuge, and then perform RT reaction.
(4) 9μl aliquots of 1st_PCR_Mix in a new 96 wells plate, add 1μl RT product to each well, and then perform 1st PCR Reaction.
(5) After PCR is completed, the 1st product is ten-fold diluted, and 1μl is used as a template for the next round of PCR.
(6) 9μl aliquot 2nd_PCR_Mix in a new 96 wells plate, add 1μl 1st diluted product to each well, and then perform 2nd PCR Reaction.
(7) Prepare 2% agar gel, load 3μl sample to analysis PCR resμlt.

Section 02. PCR Program Setup

1. For RT Reaction

45°	45min
70°	15min
4°	infinity

2. For 1st PCR Reaction

95	3min	
95	10s	30cycles
55	5s	
72°	1min	
72°	5min	
4°	infinity	

3. For 2nd PCR Reaction

95	3min	
95	10s	35cycles
55	5s	
72°	45s	
72°	5min	
4°	infinity	

Supplementary Table 7. Preparation of BCR Cloning primers

**Step 01. Dissolve primer powder in water**
the leader primers and J-region primers are dissolved to 100uM<br>the other primers are dissolved to 10μM
**Step 02. Preparation of BCR_RT_Primer_Mix (each 2μM)**
take 100μl G289_primer(10μM), K244_primer(10μM), L81 primer(10μM), respectively, then mix according to 1:1:1.
**Step 03. Preparation of AP_Leader_Mix (each 2μM)**
(1) For AP_G_Leader_Mix: Add 380μl water to a 1.5ml centrifuge tube, and take 20μl each of the 31 GV_N primers, and the final volume is 1000μl;<br>(2) For AP_K_Leader_Mix: Add 620μl water to a 1.5ml centrifuge tube, and take 20μl each of the 19 KV_N primers, and the final volume is 1000μl;<br>(3) For AP_L_Leader_Mix: Add 580μl water to a 1.5ml centrifuge tube, and take 20μl each of the 21 LV_N primers, and the final volume is 1000μl;
**Step 04. Preparation of IGHJ_region_Primer_Mix (each 2μM)**
Add 920μl water to a 1.5ml centrifuge tube, and take 20μl each of the 4 IGHJ_N primers, and the final volume is 1000μl;
**Step 05. Preparation of K194_Primer_Mix**
take 100μl each of the following 2 primers, then mix:<br>K194-primer-01 (10μM)<br>K194-primer-02 (10μM)
**Step 06. Preparation of L19_Primer_Mix**
take 100μl each of the following 3 primers, then mix:<br>L19-primer-01 (10μM)<br>L19-primer-02 (10μM)<br>L19-primer-03 (10μM)
