# Supplementary material for: A Rapid and Efficient Screening System for Neutralizing Antibodies and Its Application for SARS-CoV-2
Source: Front Immunol. 2021 Mar 22;12:653189. doi: 10.3389/fimmu.2021.653189 (PMC8019923; doi:10.3389/fimmu.2021.653189)
Supplement: Supplementary file 2 [file DataSheet_2.pdf]

Supplementary Materials for

A rapid and efficient screening system and its application for the discovery of potent neutralizing antibodies to SARS-CoV-2

Xiaojian Han, Yingming Wang, Shenglong Li, Chao Hu, Tingting Li, Chenjian Gu, Kai Wang, Meiying Shen, Jianwei Wang, Jie Hu, Ruixin Wu, Song Mu, Fang Gong, Qian Chen, Fengxia Gao, Jingjing Huang, Yingyi Long, Feiyang Luo, Shuyi Song, Shunhua Long, Yanan Hao, Luo Li, Yang Wu, Wei Xu, Xia Cai, Qingzhu Gao, Guiji Zhang, Changlong He, Kun Deng, Li Du, Yaru Nai, Wang Wang, Da Pang, Youhua Xie, Di Qu, Ailong Huang, Ni Tang[*] and Aishun Jin[*]

[*]Correspondence: aishunjin@cqmu.edu.cn (A.S.J); nitang@cqmu.edu.cn (N.T.).

Includes:

Supplementary Figure 1 to 6

Supplementary Table 1 to 7

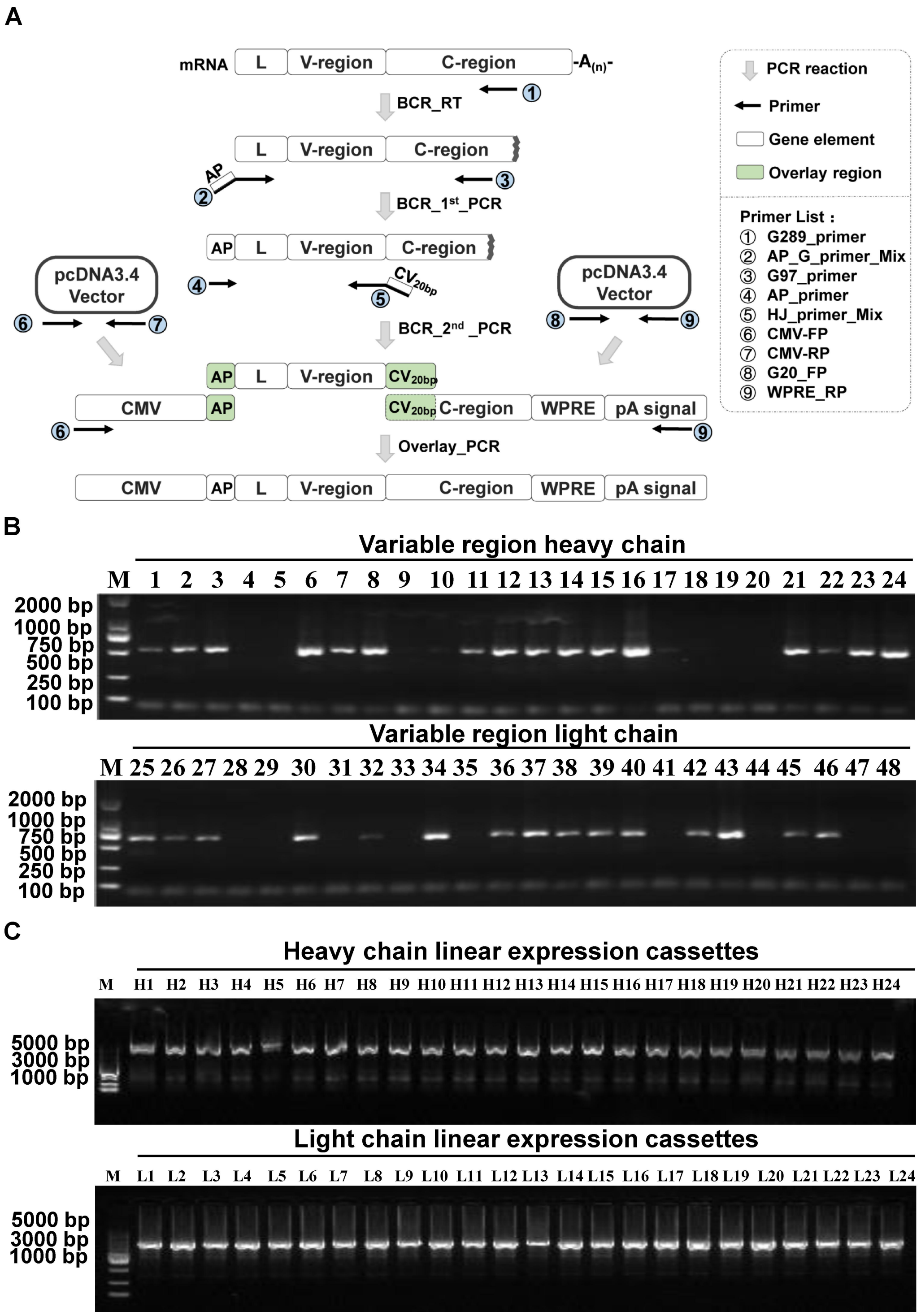

Supplementary Figure 1 | Schematic diagram of the single-cell BCR Cloning and the construction of linear expression cassettes. **(A)** The workflow of single-cell BCR Cloning and construction of linear expression cassettes. The BCR cDNAs were obtained from the single RBD-specific mB cells by RT-PCR. the variable region of the Ab gene was amplified via the 1st and 2nd PCR, and the linear expression cassettes were constructed via the 3rd overlay PCR. 1st PCR utilized gene-specific primers at both the 5′ and 3′ ends. The 5′ forward primer including an "adapter" bound to the leader sequence (L) of Ab cDNA. The 3′ reverse primer bound to the heavy or light constant regions. In the 2nd PCR, the 5′ forward primer annealed to an "adapter" locating at the 5′ end of the 1st PCR product, and the 3′ reverse primer annealed to the J gene of the Ab variable region. The 2nd PCR product provided 20 base-pair overlap regions at both sites: its 5′ end overlapping with the 3′ terminal of the human cytomegalovirus (CMV)

promoter fragment and its 3′ end overlapping with the 5′ terminal of the heavy or light chain constant region fragment, which contained a polyadenylation signal. Through the 3rd Overlay PCR, the CMV promoter fragment, the variable region fragment, and the constant region fragment were fused and amplified to produce the linear expression cassettes. **(B)** Agarose gel of BCR Cloning 2nd PCR product. The PCR products were electrophoresed and stained with ethidium bromide. Lane "M", 2 kb DNA ladder; Lane 1-24, the product of heavy chain variable region and Lane 25-48, the product of light chain variable region. **(C)** The successfully paired BCR variable region gene products were selected and constructed into a linear expression cassette. Agarose gel of 3rd PCR product. Lane "M", 5 kb DNA ladder; Lane H1-H24, linear expression cassettes of the heavy chains and Lane L1-L24, linear expression cassettes of the light chains.

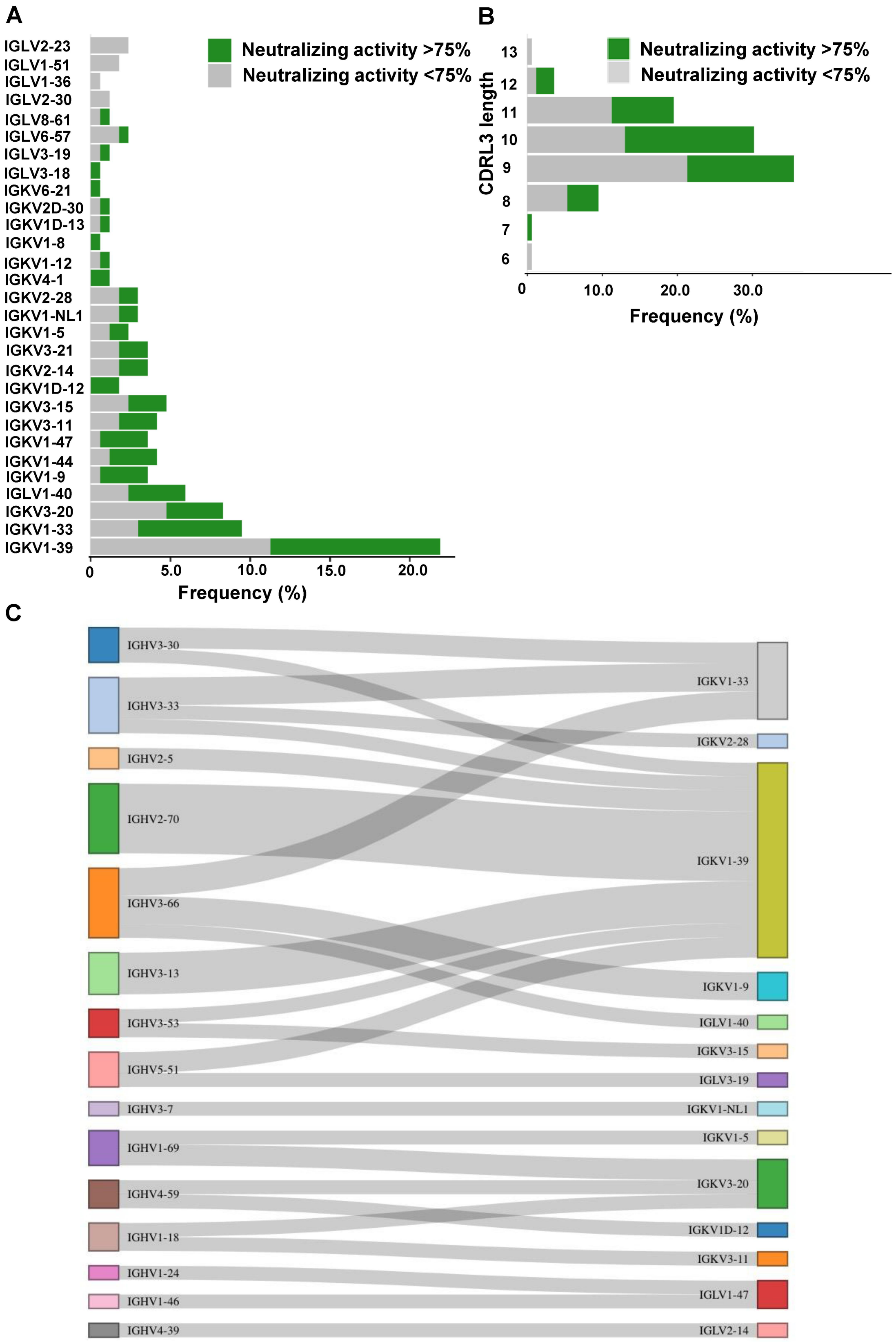

Supplementary Figure 2 | Usage of light chains, and pairing of heavy and light chains for all specific antibodies. **(A)** Usage frequencies of the variable region gene clusters of the light chain (VL) for the pote-NAbs and non-neutralizing Abs. **(B)** Frequencies of the light chain complementarity determining region 3 (CDRL3) lengths of the pote-NAbs and non-neutralizing Abs. **(C)** Variable region of Heavy and light chain were paired and highlighted in different colors.

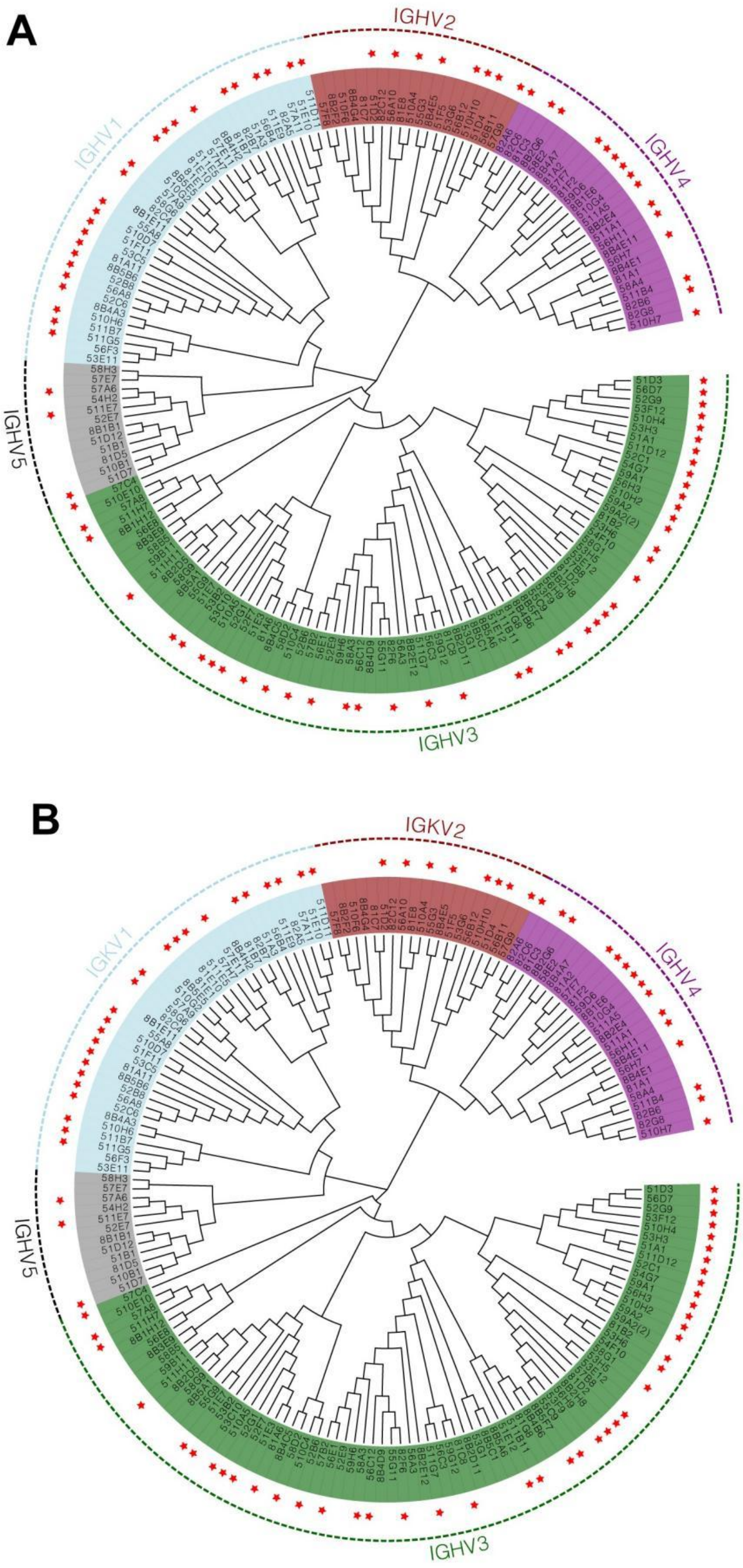

Supplementary Figure 3 | Gene family usage and phylogenetic analysis of VH and VL genes for RBD-specific antibodies. phylogenetic analysis of VH **(A)** and VL **(B)** were performed and each gene family were highlighted in different colors. The red stars represented individual neutralizing antibodies. Branch lengths were drawn to scale so that related sequences could be assessed.

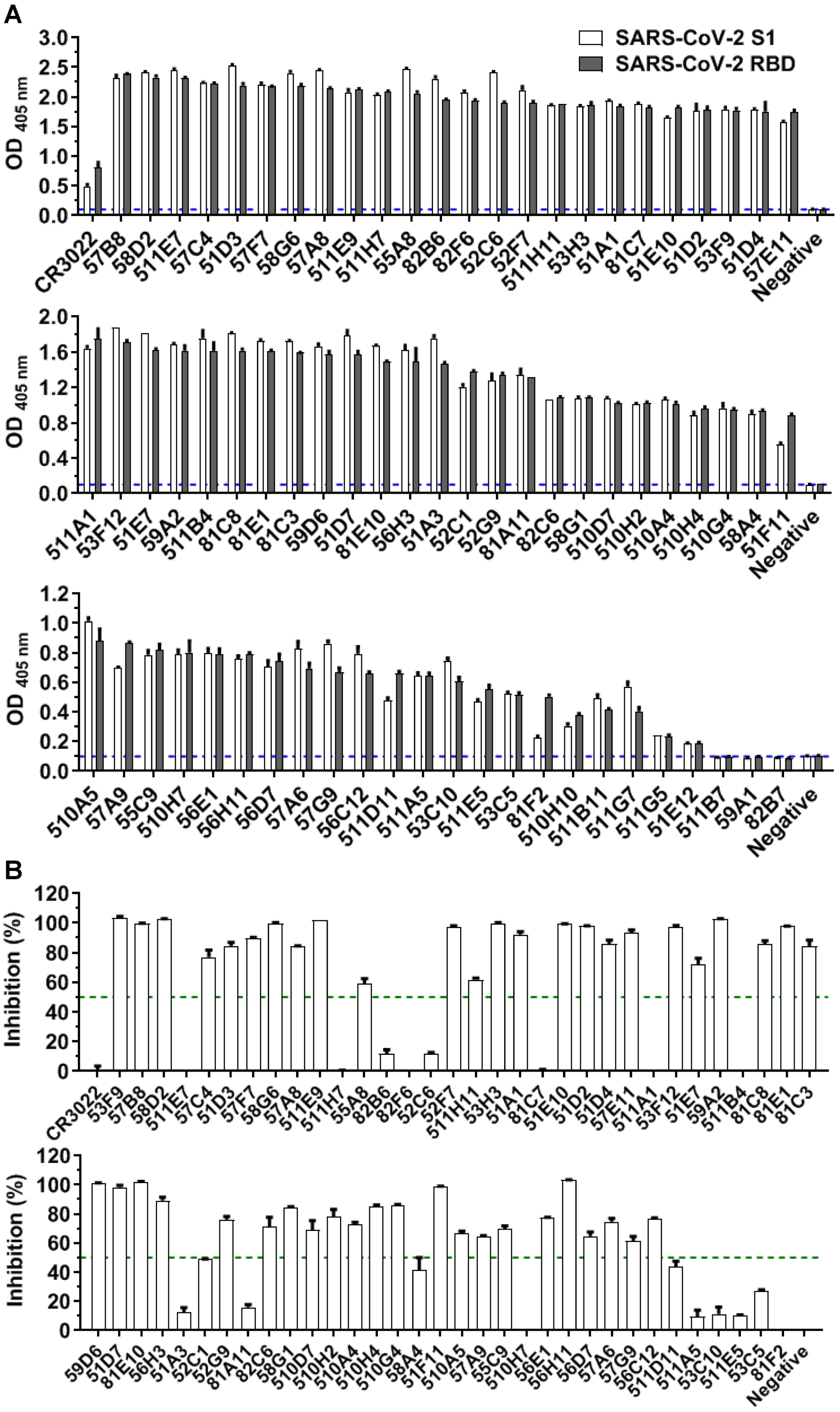

Supplementary Figure 4 | The binding activity and inhibition of ACE2-RBD interaction of mAbs tested by ELISA and competitive ELISA. **(A)** The $OD_{405\ nm}$ value refected the binding strength of purified mAbs to SARS-CoV-2 S1 or RBD. Plates were coated with 1 μg/ml recombinant S1 or RBD protein of SARS-CoV-2. A SARS specific mAb (CR3022) was set as the positive control. The blue dashed lines indicated the OD_{405nm} value of a negative sample. **(B)** The inhibitory effect of purified mAbs against the interaction between SARS-CoV-2 RBD and hACE2 was tested via competitive ELISA analysis. Blocking efficacy was determined by comparing response units with and without prior antibody incubation. The green dashed lines indicated 50% inhibition on blocking the interaction ACE2 and RBD interaction. Data were shown as mean ± SD of representative experiments.

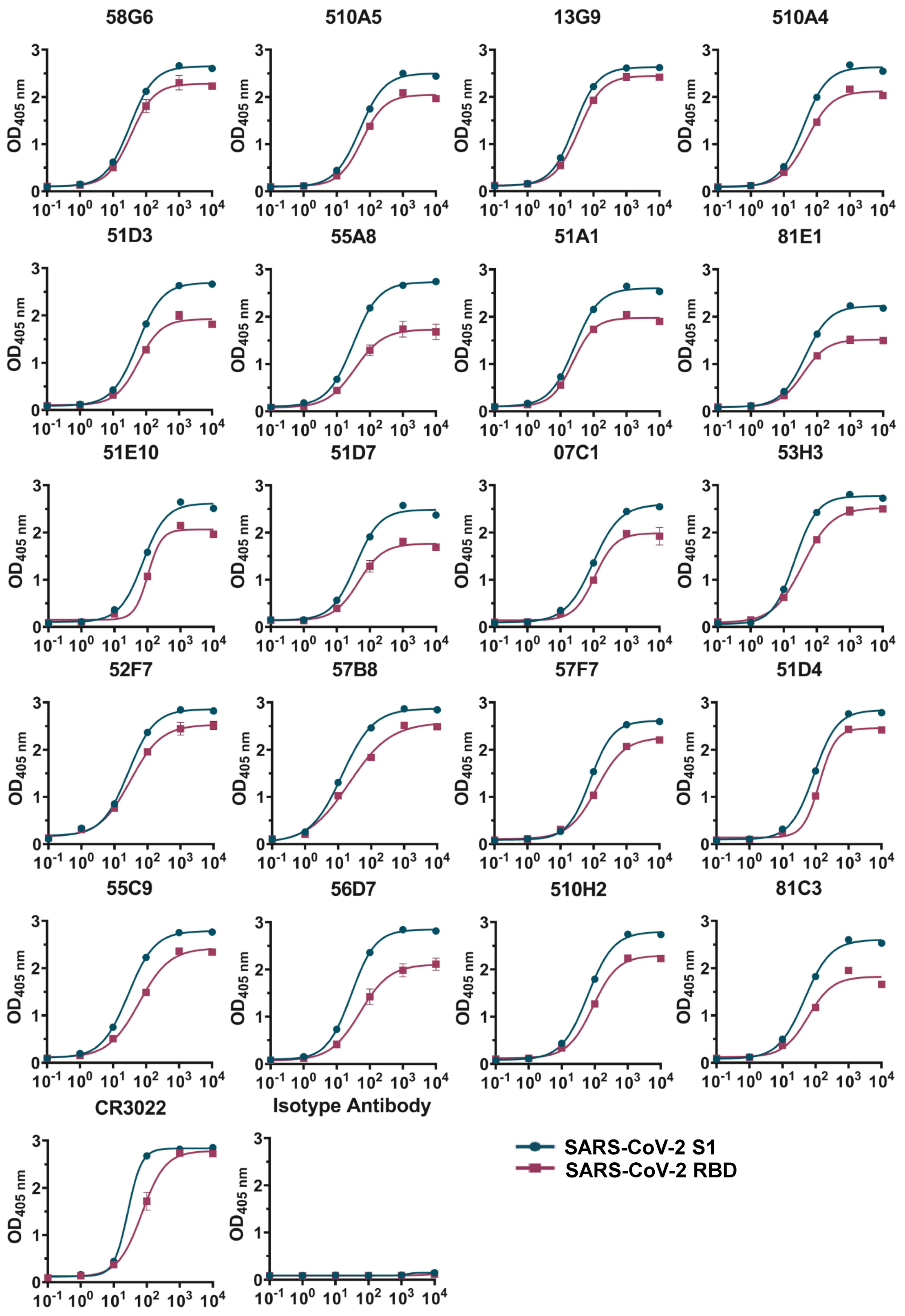

Supplementary Figure 5 | The association ability of the top 20 NAbs to SARS-CoV-2 S1 or SARS-CoV-2 S-RBD. The binding ability of the NAbs to SARS-CoV-2 S1 and S-RBD were tested with various concentrations of NAb via ELISA. The SARS-CoV specific mAb (CR3022) and the HBV antibody (Isotype antibody) were used as positive and negative control, respectively. Data are performed in technical duplicate and representative of at least 2 independent experiments. The mean ± SEM of duplicates are shown.

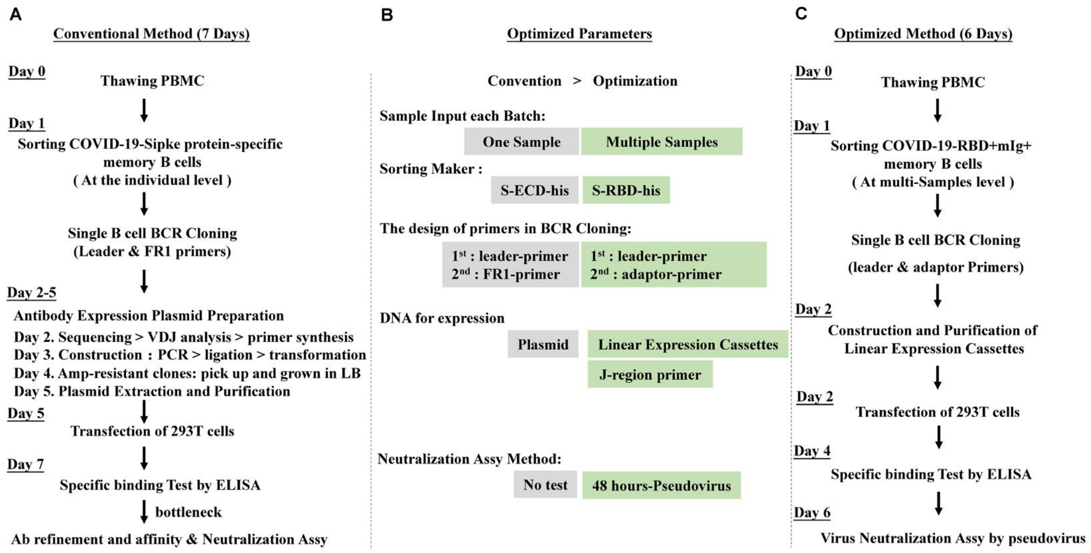

Supplementary Figure 6 | The optimization of the screening platform. **(A)** The conventional screening of neutralizing antibodies. Antigen-specific B cells from PBMCs were sorted on day 1. The single-cell BCR genes were amplified by PCR. The antibody expression vectors were constructed in the next four days, including the PCR product sequencing, the primer synthesis, the ligation of genes and vectors, the plasmid transformation and the plasmid extraction. The purified plasmids were transfected into HEK293T cells on day 5. After 48 hours, the cell supernatants were collected and used to analysis the specific binding ability by ELISA. Specific antibodies are used for following antibody expression and purification. Purified antibodies were screened for neutralizing capability. **(B)** The key parameters affecting screening efficiency. The following steps of the screening processes were carefully modified: sorting the individual sample or the pooled samples, labeling S or just RBD specific memory B cells, designing optimized primers for the single-cell BCR cloning, expressing antibodies using plasmids or linear expression cassettes. To reduce time-consuming and workload, it is the critical step to screen neutralizing antibodies during the initial screening in the sixty days. **(C)** The optimized strategy of neutralizing antibodies screening. One day after PBMC thawing, specific B cell sorting was performed and single-cell BCR genes were cloned on day 1. The 2nd PCR products were used to construct the linear expression cassettes, which were transfected into HEK293T cells, instead of constructing plasmid. After 48 hours, the supernatants of each transfected samples were harvested and analyzed the specific binding via ELISA. The pseudovirus neutralization assay was evaluated on day6.
